# Safety and Immunogenicity of a Messenger RNA–Based Cytomegalovirus Vaccine in Healthy Adults: Results From a Phase 1 Randomized Clinical Trial

**DOI:** 10.1093/infdis/jiae114

**Published:** 2024-03-13

**Authors:** Carlos Fierro, Daniel Brune, Marian Shaw, Howard Schwartz, Conor Knightly, Jiang Lin, Andrea Carfi, Andrew Natenshon, Shiva Kalidindi, Caroline Reuter, Jacqueline Miller, Lori Panther

**Affiliations:** Johnson County Clin-Trials, Department of Clinical Safety & Risk Management, Lenexa, Kansas; Optimal Research, LLC, Peoria, Illinois; Advanced Clinical Research, Meridian, Idaho; Research Centers of America, LLC, Hollywood, Florida; Moderna, Inc, Department of Clinical Development Operations, Cambridge, Massachusetts; Moderna, Inc, Department of Biostatistics, Cambridge, Massachusetts; Moderna, Inc, Department of Research and Development, Cambridge, Massachusetts; Moderna, Inc, Department of Infectious Disease Development, Cambridge, Massachusetts; Moderna, Inc, Department of Statistical Programming, Cambridge, Massachusetts; Johnson County Clin-Trials, Department of Clinical Safety & Risk Management, Lenexa, Kansas; Moderna, Inc, Department of Infectious Diseases, Cambridge, Massachusetts; Moderna, Inc, Department of Infectious Diseases, Cambridge, Massachusetts

**Keywords:** cytomegalovirus, CMV, congenital cytomegalovirus, cCMV, viral vaccine

## Abstract

**Background:**

This phase 1 trial evaluated the safety, reactogenicity, and immunogenicity of mRNA-1647, a messenger RNA (mRNA)–based cytomegalovirus (CMV) vaccine, in CMV-seronegative and -seropositive adults.

**Methods:**

Participants were randomly assigned to receive 30, 90, 180, or 300 µg of mRNA-1647 or placebo on a 0-, 2-, and 6-month schedule and followed for 12 months after the last dose.

**Results:**

A total of 154 (80 CMV-seronegative and 74 CMV-seropositive) participants were enrolled; 118 participants were randomized to mRNA-1647 and 36 to placebo. Mean (standard deviation) age was 32.5 (8.6) and 35.1 (8.9) years in the placebo and mRNA-1647 groups, respectively, in phase B (63% and 64% female) and 42.5 (6.2) and 33.3 (8.7) years, respectively, in phase C (2% and 16% female). No deaths, related serious adverse events, or adverse events of special interest were reported. Most adverse reactions were grade ≤2 severity. Increased neutralizing antibody, binding antibody, and antigen-specific cell-mediated responses were observed across mRNA-1647 treatment groups, regardless of CMV serostatus.

**Conclusions:**

This phase 1, first-in-human trial demonstrated that mRNA-1647 has an acceptable safety profile in adults and elicits humoral and cellular immune responses.

***Clinical Trials Registration.*** NCT03382405.

## Teaser

The mRNA-based cytomegalovirus (CMV) vaccine, mRNA-1647, was evaluated at the 30-, 90-, 180-, and 300-μg dose levels in a phase 1 trial in CMV-seronegative and CMV-seropositive adults. mRNA-1647 had an acceptable safety profile and elicited humoral and cellular immune responses.

## Graphical Abstract

**Graphical Abstract:**
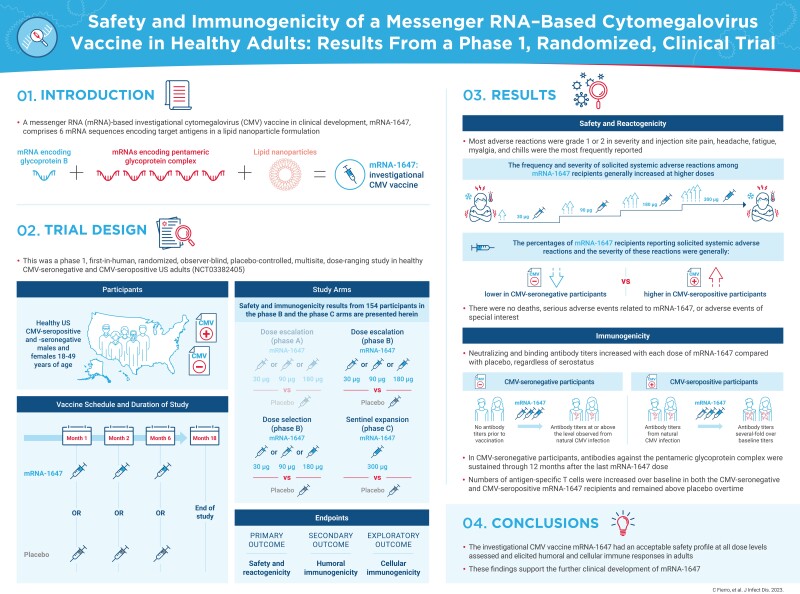

Cytomegalovirus (CMV) is a common infection that establishes lifelong latency in myeloid cells [1, 2]. Though largely asymptomatic in healthy populations, CMV infection is the source of substantial morbidity and mortality in immunosuppressed individuals and fetuses infected in utero [1–5]. CMV is the most common congenitally acquired infection and nongenetic cause of sensorineural hearing loss diagnosed in infancy and childhood [6]. In immunosuppressed individuals such as solid organ and hematopoietic stem-cell transplant recipients, CMV infection is an important contributor to graft rejection and mortality [5, 7]. A vaccine-based approach to prevent infection and the substantial burden of disease is a promising therapeutic option; however, despite several decades of effort, an approved CMV vaccine remains elusive [8].

The CMV glycoprotein B (gB) and pentameric glycoprotein complex (pentamer) facilitate viral entry into a variety of human cell types, including placental cytotrophoblasts, and are dominant targets of the human immune response to CMV infection [9–13]. While there is no established immunological correlate of protection against infection in CMV-seronegative populations, or against CMV reinfection or reactivation in CMV-seropositive populations, several observations indicate the importance of immunity against gB and pentamer in CMV infection and disease [9, 12].

mRNA-1647, a messenger RNA (mRNA)–based CMV vaccine candidate in clinical development, contains 6 discrete mRNA sequences formulated in lipid nanoparticles [14–16]. In murine and nonhuman primate models, mRNA-1647 elicited neutralizing antibody (nAb) titers against CMV infection in both epithelial cells and fibroblasts [14]. mRNA-1647 is the first mRNA-based CMV vaccine evaluated in humans and represents a milestone in mRNA vaccine technology by demonstrating that 5 discrete mRNA sequences encoding the complex multimeric pentamer antigen can be delivered, expressed, assembled, and presented in a manner analogous to that of natural CMV infection [14].

The objective of this phase 1 first-in-human trial was to assess safety, reactogenicity, and immunogenicity of different dose levels of the investigational CMV vaccine mRNA-1647 administered according to a 3-dose schedule in healthy CMV-seronegative and CMV-seropositive participants 18–49 years of age.

## METHODS

### Trial Design

This phase 1, randomized, observer-blind, placebo-controlled, dose-ranging, first-in-human trial was conducted at 4 US sites from November 2017 to October 2020 (NCT03382405). The trial evaluated mRNA-1647 in consecutive phases (Supplementary Figure 1). Dose-escalation phase A sequentially enrolled CMV-seronegative participants into 3 dose-level arms (mRNA-1647 30 µg, 90 µg, or 180 µg), with each arm randomized 4:1 to receive either mRNA-1647 or saline placebo; dose-escalation phase B evaluated mRNA-1647 manufactured under a modified process and sequentially enrolled CMV-seronegative participants into 3 dose-level arms (mRNA-1647 30 µg, 90 µg, or 180 µg), with each arm randomized 4:1 to receive either mRNA-1647 or saline placebo; dose-selection phase B enrolled CMV-seronegative and CMV-seropositive participants randomly assigned in parallel 1:1:1:1 to receive either mRNA-1647 30 µg, 90 µg, or 180 µg or saline placebo; and sentinel-expansion phase C enrolled CMV-seronegative and CMV-seropositive participants randomly assigned 4:1 to receive either mRNA-1647 300 µg or saline placebo. Injections were administered on a 3-dose schedule at day 1, month 2, and month 6, and participants were followed for 12 months after the last injection.

Different dose levels of a separate investigational CMV vaccine encoding for a phosphorylation-deficient mutant of the CMV pp65 protein (mRNA-1443) were evaluated in 12 participants in dose-escalation phase A. Dose-escalation phase A evaluated safety and reactogenicity only; data are reported in Supplementary Tables 1–4 and the Supplementary Results. Safety and immunogenicity results from the phase B treatment groups (mRNA-1647 30 µg, 90 µg, or 180 µg or placebo) and the phase C treatment groups (mRNA-1647 300 µg or placebo) are presented herein.

The trial was observer-blind with blinding of investigators, site personnel, and sponsor. Unblinded personnel who had no other role in the trial conduct prepared and administered the injections in a manner that shielded the participant and blinded site personnel from viewing the dose. Participants were assigned to treatment groups using an interactive response technology system. An internal safety team and independent safety monitoring committee provided oversight.

The Advarra institutional review board reviewed and approved the protocol, amendments, and informed consent form. All participants provided written informed consent before trial entry and performance of procedures. The trial was conducted according to the protocol; applicable national, state, and local laws or regulations; and the principles of the International Council for Harmonisation harmonized tripartite guideline E6(R2): Good Clinical Practice and in the Declaration of Helsinki.

### Outcomes

The primary objective was to evaluate the safety and reactogenicity of different dose levels of mRNA-1647 administered according to a 3-dose schedule. Secondary objectives were to evaluate anti-CMV nAb responses against epithelial cell infection (a measure of anti-pentamer response) and against fibroblast infection (a measure of anti-gB response) and antigen-specific binding immunoglobulin G (IgG) responses following mRNA-1647. An exploratory objective was to evaluate antigen-specific T-cell responses to mRNA-1647.

### Participants

Participants were healthy adults 18–49 years of age with a body mass index of 18–35 kg/m^2^. Full inclusion/exclusion criteria are shown in the Supplementary Methods. CMV serostatus at screening was determined by presence or absence of anti-CMV IgG in serum (Elecsys CMV IgG; Roche Diagnostics).

### Trial Injections

mRNA-1647 was provided as a sterile liquid for injection at concentrations of 1.0 or 2.0 mg/mL and was prepared by diluting the appropriate concentration of mRNA-1647 with sterile saline to result in a dose volume of 0.5 mL for each dose level. Placebo was provided as 0.9% sodium chloride injection (USP; normal saline) and administered at a volume of 0.5 mL. Injections were administered intramuscularly into the deltoid muscle.

### Safety and Reactogenicity

Safety (events occurring at any time) and reactogenicity (events occurring soon after trial injection) assessments included solicited local and systemic adverse reactions (ARs), unsolicited adverse events (AEs), medically attended AEs (MAAEs), serious AEs (SAEs), AEs of special interest (AESIs; Supplementary Methods), and AEs leading to withdrawal. Solicited ARs and unsolicited AEs were collected through 7 and 28 days after each injection, respectively. MAAEs, SAEs, and AESIs were collected throughout the trial.

### Immunogenicity

Blood samples for antibody-mediated immunogenicity assessments were collected on day 1 and months 1, 3, 6, 7, 12, and 18. Outcomes included nAb titers against epithelial cell infection and against fibroblast infection, and binding IgG titers against the gB antigen and against the pentamer antigen. Cell-based neutralizing assays were used to measure nAb titers (Supplementary Methods). Binding IgG titers were measured by enzyme-linked immunosorbent assay (Supplementary Methods).

Blood samples were collected on days 1, 63, 175, and 337 (month 12) for cell-mediated immunogenicity. Outcomes included number of antigen-specific T cells secreting interferon gamma (IFN-γ) reported as spot-forming cells/10^6^ peripheral blood mononuclear cells, as determined by enzyme-linked immunospot assay (ELISpot) using the Human IFN-γ ELISpot^PRO^ Kit (Mabtech) and glycoprotein H (gH) and gB peptide pools (Genscript).

### Statistical Analysis

The sample size was sufficient to provide a descriptive summary of the safety and immunogenicity of mRNA-1647. Sample size was not driven by statistical assumptions for formal hypothesis testing because previous clinical data were unavailable and clinically meaningful group differences were not established. Analyses were performed using SAS version 9.4 software (SAS Institute, Cary, North Carolina).

Data are presented by CMV serostatus and treatment group. Data from the mRNA-1647 treatment groups in dose-escalation phase B and dose-selection phase B (30 µg, 90 µg, and 180 µg mRNA-1647) were combined for all outcomes. Immunogenicity data from the phase B and C placebo groups were pooled. Data analysis sets are defined in the Supplementary Methods.

Categorical variables were summarized using frequencies and percentages, and continuous variables were summarized using descriptive statistics. For antibody-mediated immunogenicity, geometric mean titers (GMTs) and 95% confidence intervals (CIs) were provided at each timepoint. Geometric mean ratios (GMRs) and 95% CIs at each individual post-dose timepoint over baseline were provided for CMV-seropositive participants. The 95% CIs were calculated based on the *t*-distribution of log-transformed values (GMTs) or the difference in the log-transformed values (GMRs) and back-transformed to the original scale for presentation. For cell-mediated immunogenicity, means and standard deviations (SDs) for the observed values and changes from baseline were provided at each timepoint.

## RESULTS

### Participants

**Figure 1.**
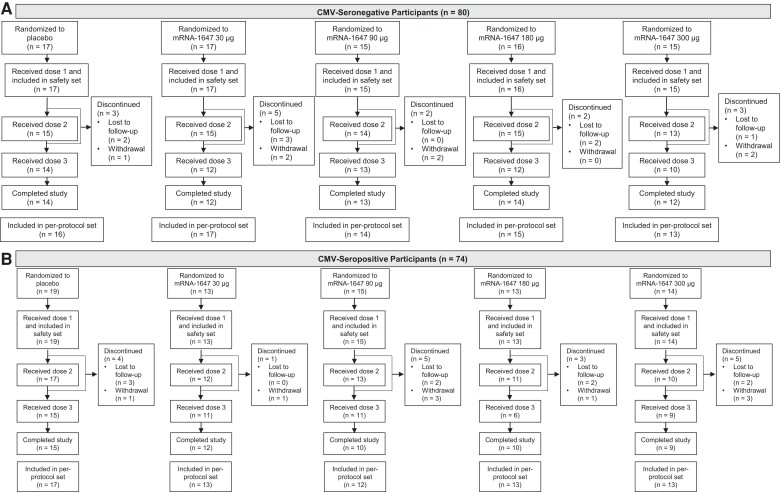
Participant disposition by cytomegalovirus (CMV) serostatus. The flow of CMV-seronegative (*A*) and CMV-seropositive (*B*) participants by treatment group. Data are from the randomized set. Participants who received ≥1 dose were included in the safety set. Participants in the per-protocol set had no major protocol deviations, received vaccinations per schedule, and complied with timing of the postvaccination blood sampling. Discontinued indicates discontinuation from the trial.

This study included 154 participants (61% female, 39% male) enrolled in phases B and C (80 CMV-seronegative and 74 CMV-seropositive): 118 (63 CMV-seronegative, 55 CMV-seropositive) and 36 participants (17 CMV-seronegative, 19 CMV-seropositive), respectively. Participant disposition is presented in Figure 1. Participants were randomly assigned to receive mRNA-1647 or placebo: 30 (17 CMV-seronegative and 13 CMV-seropositive) received 30 μg, 30 (15 CMV-seronegative and 15 CMV-seropositive) received 90 μg, 29 (16 CMV-seronegative and 13 CMV-seropositive) received 180 μg, 29 (15 CMV-seronegative and 14 CMV-seropositive) received 300 μg, and 36 (17 CMV-seronegative and 19 CMV-seropositive) received placebo.

Overall demographics are presented in Table 1, and demographics by serostatus are presented in Supplementary Table 5. In phase B, mean age of participants assigned to receive mRNA-1647 or placebo was 35.1 (SD, 8.9) and 32.5 (SD, 8.6) years, respectively, and most were female (64.0% and 63.3%, respectively) and White (87.6% and 80.0%). In phase C, mean age of participants assigned to receive mRNA-1647 or placebo was 33.3 (SD, 6.2) and 42.5 (SD, 8.7) years, respectively; most were female (55.2%) and White (82.8%) in the mRNA-1647 group and male (66.7%) and White (83.3%) in the placebo group.

**Table 1.** Participant Demographics Overall^a^

Characteristic	Phase B					Phase C	
	Placebo (n = 30)^b^	mRNA-1647				Placebo (n = 6)^b^	mRNA-1647
		30 µg (n = 30)^b^	90 µg (n = 30)^b^	180 µg (n = 29)^b^	Total mRNA (n = 89)		300 µg (n = 29)^b^
Sex, No. (%)^c^							
Male	11 (36.7)	10 (33.3)	12 (40.0)	10 (34.5)	32 (36.0)	4 (66.7)	13 (44.8)
Female	19 (63.3)	20 (66.7)	18 (60.0)	19 (65.5)	57 (64.0)	2 (33.3)	16 (55.2)
Age, y, mean (SD)	32.5 (8.6)	36.0 (8.0)	35.4 (8.5)	34.0 (10.1)	35.1 (8.9)	42.5 (6.2)	33.3 (8.7)
Race, No. (%)^c^							
White	24 (80.0)	28 (93.3)	24 (80.0)	26 (89.7)	78 (87.6)	5 (83.3)	24 (82.8)
Black/African American	5 (16.7)	2 (6.7)	6 (20.0)	2 (6.9)	10 (11.2)	1 (16.7)	4 (13.8)
Asian	0	0	0	1 (3.4)	1 (1.1)	…	…
American Indian or Alaska Native	1 (3.3)	0	0	0	0	…	…
Not reported	…	…	…	…	…	0	1 (3.4)
Ethnicity, No. (%)^c^							
Not Hispanic or Latino	27 (90.0)	29 (96.7)	30 (100.0)	29 (100.0)	88 (98.9)	5 (83.3)	20 (69.0)
Hispanic or Latino	3 (10.0)	1 (3.3)	0	0	1 (1.1)	1 (16.7)	9 (31.0)
CMV serostatus at screening, No. (%)^c^							
Negative	16 (53.3)	17 (56.7)	17 (56.7)	16 (55.2)	50 (56.2)	3 (50.0)	15 (51.7)
Positive	14 (46.7)	13 (43.3)	13 (43.3)	13 (44.8)	39 (43.8)	3 (50.0)	14 (48.3)
CMV serostatus at baseline, No. (%)^c,d^							
Negative	14 (46.7)	17 (56.7)	15 (50.0)	16 (55.2)	48 (53.9)	3 (50.0)	15 (51.7)
Positive	16 (53.3)	13 (43.3)	15 (50.0)	13 (44.8)	41 (46.1)	3 (50.0)	14 (48.3)

Abbreviations: CMV, cytomegalovirus; mRNA, messenger RNA; nAb, neutralizing antibody; SD, standard deviation.

^a^Data are from the exposed set, which consisted of all participants in the randomized set who received any injection.

^b^Number of participants exposed to placebo or mRNA-1647.

^c^Number of participants exposed to placebo or mRNA-1647 in category with nonmissing data.

^d^CMV serostatus at baseline was considered positive if either the nAb against epithelial cell infection or against fibroblast infection was above the lower limit of quantification at baseline.

### Solicited Local Adverse Reactions

**Figure 2.**
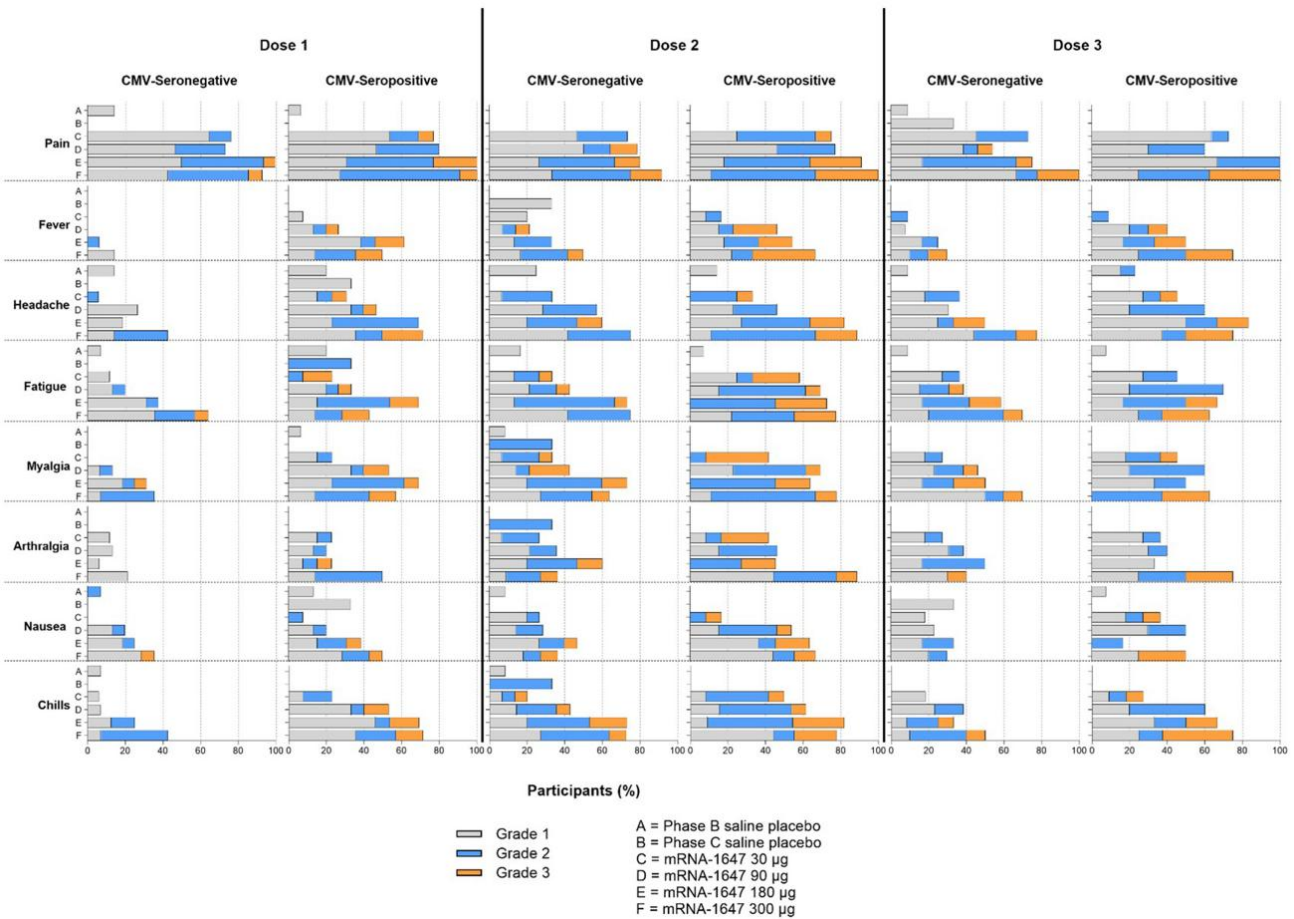
Solicited local and systemic adverse reactions (ARs) in cytomegalovirus (CMV)–seronegative and -seropositive participants by treatment group and grade following each dose. Data are from the dose 1, dose 2, and dose 3 solicited safety sets. As depicted in the key, placebo data are reported separately for phase B (mRNA-1647 30 μg, 90 μg, or 180 μg or saline placebo) and phase C (mRNA-1647 300 μg or saline placebo).

Solicited local ARs are presented by serostatus in Figure 2 and Supplementary Tables 6 and 7. In phase B overall, local ARs were reported in the mRNA-1647 groups by 83.3%, 77.3%, and 66.7% of CMV-seronegative participants and by 85.4%, 80.6%, and 74.1% of CMV-seropositive participants after doses 1, 2, and 3, respectively. In phase C overall, local ARs were reported by 92.9%, 91.7%, and 90.0% of CMV-seronegative participants and by 91.7%, 100%, and 100% of CMV-seropositive participants after doses 1, 2, and 3, respectively. Although overall frequencies of local ARs in CMV-seropositive participants were similar after doses 1 (88.6%), 2 (90.3%), and 3 (87.1%) in CMV-seronegative participants, frequencies were higher after dose 1 (88.1%) than after dose 2 (84.5%) or 3 (78.4%). Injection site pain was most frequent in the mRNA-1647 groups, reported by 83.3%, 77.3%, and 66.7% of CMV-seronegative phase B participants and by 92.9%, 91.7%, and 100% of CMV-seronegative phase C participants after doses 1, 2, and 3, respectively, and by 85.4%, 80.6%, and 74.1% of CMV-seropositive phase B participants and 100.0%, 100%, and 100% of CMV-seropositive phase C participants after doses 1, 2, and 3, respectively. In both the CMV-seronegative and CMV-seropositive cohorts, higher proportions of participants in the 180-μg (100%, 80.0%, and 75.0% of CMV-seronegative and 100%, 90.9%, and 100% of CMV-seropositive participants after doses 1, 2, and 3, respectively) and 300-μg (92.9%, 91.7%, and 100% of CMV-seronegative and 100%, 100%, and 100% of CMV-seropositive participants after doses 1, 2, and 3, respectively) mRNA-1647 groups reported injection site pain compared with the 30-μg (76.5%, 73.3%, and 72.7% of CMV-seronegative and 76.9%, 75.0%, and 72.7% of CMV-seropositive participants after doses 1, 2, and 3, respectively) and 90-μg (73.3%, 78.6%, and 53.8% of CMV-seronegative and 80.0%, 76.9%, and 60.0% of CMV-seropositive participants after doses 1, 2, and 3, respectively) mRNA-1647 groups. Except for injection site pain, local ARs were generally more common after dose 2 and 3 and among CMV-seronegative participants for each dose level.

### Solicited Systemic Adverse Reactions

Solicited systemic ARs are presented by serostatus in Figure 2 and Supplementary Tables 6 and 7. Overall, systemic ARs were reported more frequently and were of higher severity in the CMV-seropositive mRNA-1647 groups compared to CMV-seronegative mRNA-1647 groups. Systemic ARs were reported by 37.5%, 65.9%, and 61.1% of CMV-seronegative phase B participants and by 78.6%, 91.7%, and 90.0% of CMV-seronegative phase C participants after doses 1, 2, and 3, respectively, compared to 70.7%, 75.0%, and 66.7% of CMV-seropositive phase B participants and 92.9%, 100%, and 100% of CMV-seropositive phase C participants after doses 1, 2, and 3, respectively. Headache, fatigue, myalgia, and chills were most frequently reported. The frequency and severity of systemic ARs generally increased with increasing dose level.

### Unsolicited Adverse Events

Unsolicited AEs are presented in Supplementary Tables 3 and 4. No deaths, SAEs related to treatment, or AESIs were reported. Unsolicited AEs were reported by similar proportions of participants in the mRNA-1647 and placebo groups. Reports of most treatment-related unsolicited AEs in the mRNA-1647 groups were consistent with solicited local or systemic ARs or other local injection site reactions. Treatment-related unsolicited MAAEs were reported by 1 CMV-seropositive participant in the mRNA-1647 180-μg group (grade 2 urticaria) and 3 participants in the mRNA-1647 300-μg group (grade 2 dizziness in a CMV-seronegative participant, grade 2 arthralgia in a CMV-seronegative participant, and grade 2 gastroenteritis in a CMV-seropositive participant). All SAEs were assessed as unrelated to treatment: 1 SAE of endometriosis was reported in a CMV-seropositive participant in the mRNA-1647 300-μg group, and SAEs of eyelid injury, road traffic accident, skin laceration, sacral and pelvic fractures, concussion, depression, and suicide attempt were reported in 1 CMV-seronegative placebo recipient. Although no AEs leading to trial withdrawal occurred, treatment-related AEs leading to discontinuation from trial injection were reported in 11 participants and were nonserious events (Supplementary Tables 3 and 4).

### Antibody-Mediated Immunogenicity

**Figure 3.**
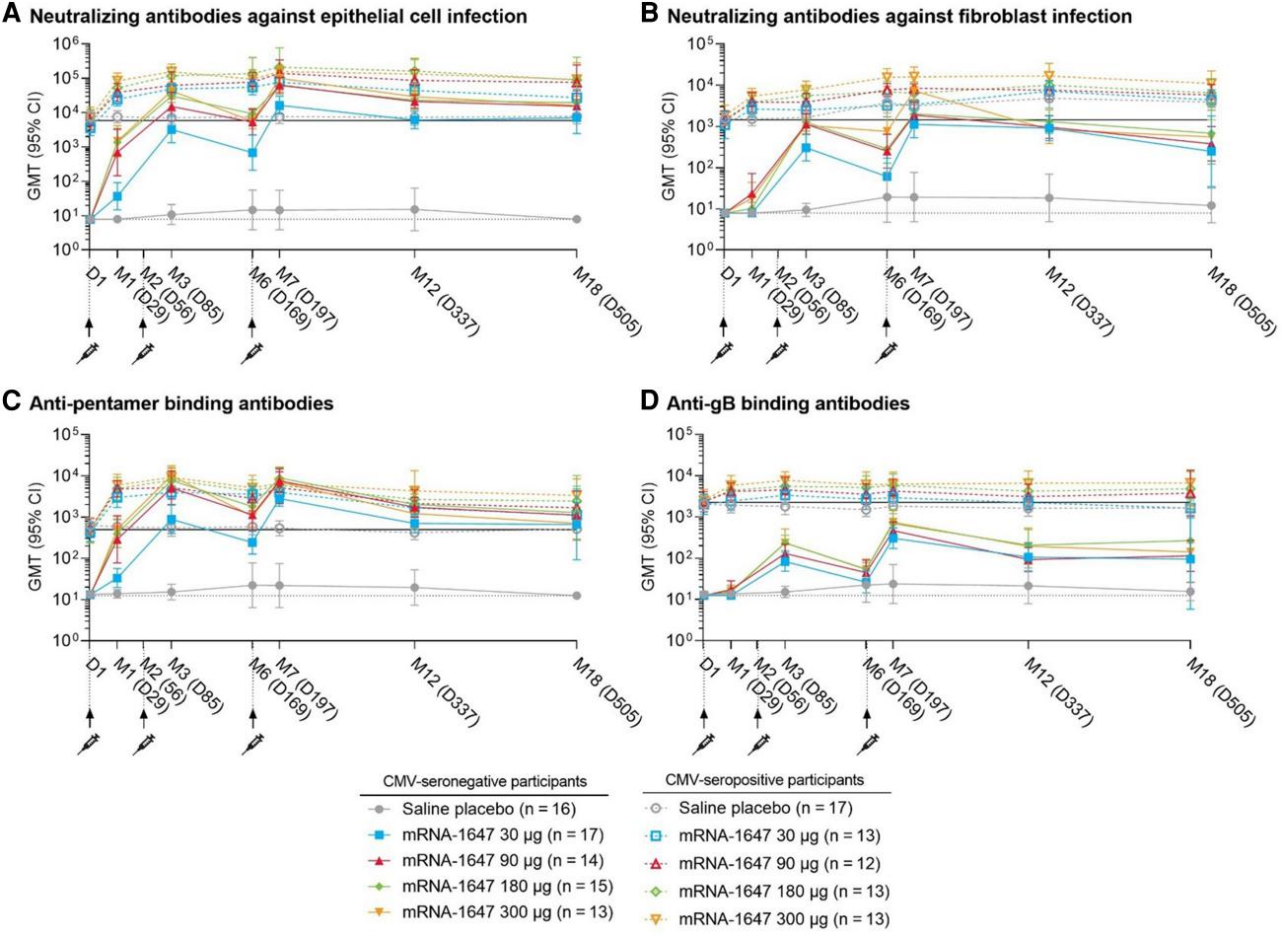
Antibody-mediated immunogenicity by cytomegalovirus (CMV) serostatus. Data are presented by serostatus, treatment group, and visit as geometric mean titer (GMT) and corresponding 95% confidence interval (CI). Data for neutralizing antibody (nAb) titers against epithelial cell infection (*A*), nAb titers against fibroblast infection (*B*), anti-pentamer binding immunoglobulin G (IgG) titers (*C*), and anti-glycoprotein B (gB) binding IgG titers (*D*) are presented by CMV serostatus from day (D) 1 through month (M) 18. CMV-seronegative (solid lines) and CMV-seropositive (dashed lines) treatment groups were plotted over time. The first, second, and third injections were administered on D1, M2, and M6, respectively, as represented by an arrow and syringe. nAb lower limit of quantification (LLOQ) = 16 and binding antibody LLOQ = 25; the dotted black lines represent half of the respective LLOQs. The solid black lines represent the baseline GMTs of the CMV-seropositive cohort (ie, CMV-seropositive GMT benchmark). Data are from the per-protocol set. n = number of participants in any per-protocol set.

Baseline GMTs in the CMV-seropositive cohort for nAbs against epithelial cell infection (5917.3) and against fibroblast infection (1448.9), anti-gB binding IgG (2250.8), and anti-pentamer binding IgG (499.4) were used to compare vaccine-induced immune responses (referred to as the CMV-seropositive benchmark; Figure 3 and Supplementary Figure 2). All dose levels of mRNA-1647 were immunogenic in both CMV-seronegative and CMV-seropositive mRNA-1647 groups. Neutralizing antibody and binding IgG GMTs generally increased after each dose and peaked at month 7. In the CMV-seronegative mRNA-1647 groups, nAb GMTs against epithelial cell infection exceeded the CMV-seropositive benchmark after dose 2 (month 3) at the 90-, 180-, and 300-µg dose levels and after dose 3 (month 7) through month 18 at all dose levels (Figure 3*A*). At month 7, nAb GMTs against fibroblast infection approximated the CMV-seropositive benchmark at the 30- and 90-µg dose levels, exceeded the CMV-seropositive benchmark at the 180- and 300-µg dose levels, then fell below the CMV-seropositive benchmark at months 12 through 18 (Figure 3*B*). Anti-pentamer binding IgG GMTs exceeded the CMV-seropositive benchmark in all mRNA-1647 groups starting after dose 2 (month 3) and remained above the CMV-seropositive benchmark through month 18 (Figure 3*C*). The anti-gB binding IgG GMTs were below the CMV-seropositive benchmark but above baseline at all dose levels and timepoints (Figure 3*D*).

In the CMV-seropositive mRNA-1647 groups, nAb GMTs against epithelial cell infection and against fibroblast infection generally increased after each dose (Figure 3*A* and 3*B*). nAb GMRs against epithelial cell infection ranged from 13.4 to 40.8 after dose 3 (month 7), and 7.0 to 14.2 at month 18 (Supplementary Table 8). nAb GMRs against fibroblast infection ranged from 4.0 to 7.1 after dose 3 (month 7) and from 3.1 to 9.5 at month 18. Anti-pentamer binding IgG GMTs generally increased after each dose in all mRNA-1647 groups, and anti-gB binding IgG GMTs increased after dose 1 and again after dose 2, and increased only in the 180-μg group after dose 3 (Figure 3*C* and 3*D*). Anti-pentamer binding IgG GMRs were highest in the 180-μg group, ranging from 8.5 to 12.5 after dose 3 (month 7) and 2.3 to 5.0 at month 18, respectively, and anti-gB binding IgG GMRs ranged from 1.9 to 2.0 and 1.3 to 1.6 after dose 3, and were generally similar across mRNA-1647 groups at months 12 and 18.

### Cell-Mediated Immunogenicity

**Figure 4.**
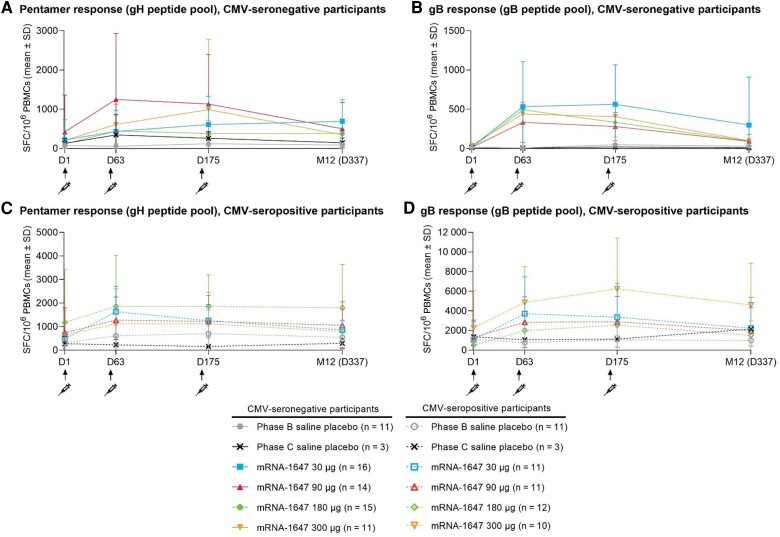
Antigen-specific cell-mediated immunogenicity by cytomegalovirus (CMV) serostatus (mean value). Data are presented by treatment group and visit through month 12 and reported as mean number ± standard deviation (SD) of antigen-specific spot-forming cells (SFC)/10^6^ peripheral blood mononuclear cells (PBMCs) as determined by interferon-γ enzyme-linked immunospot assay. *A*, Pentamer response (glycoprotein H [gH] peptide pool) in the CMV-seronegative treatment groups. *B*, Glycoprotein B (gB) response (gB peptide pool) in the CMV-seronegative treatment groups. *C*, Pentamer response (gH peptide pool) in the CMV-seropositive treatment groups. *D*, gB response (gB peptide pool) in the CMV-seropositive treatment groups. CMV-seronegative (solid lines) and CMV-seropositive (dashed lines) treatment groups were plotted over time. Doses 1, 2, and 3 were administered on day (D) 1, month (M) 2, and M6, respectively, as represented by an arrow and syringe. As depicted in the key, data are reported separately for phase B (mRNA-1647 30 μg, 90 μg, or 180 μg or saline placebo) and phase C (mRNA-1647 300 μg or saline placebo). Data are from the cell-mediated immunogenicity set.

Antigen-specific T-cell responses are presented by CMV serostatus in Figure 4, and changes from baseline are presented in Supplementary Figure 3. Mean numbers of gH- or gB-specific T cells were increased over baseline at all postbaseline timepoints in both the CMV-seronegative and CMV-seropositive mRNA-1647 groups, peaked at either day 63 or day 175 (6 days after doses 2 or 3, respectively), and remained above placebo at day 337 (month 12). A dose-dependent response trend was not apparent across mRNA-1647 groups in either serostatus cohorts. The number of gH- or gB-specific T cells remained relatively constant in the placebo groups.

## DISCUSSION

This first-in-human phase 1 trial of the investigational CMV vaccine candidate mRNA-1647 demonstrated an acceptable safety profile at dose levels ranging from 30 μg to 300 μg. In CMV-seronegative mRNA-1647 recipients, nAb and binding IgG GMTs generally increased after each dose, with generally sustained responses of nAb against epithelial cell infection through 12 months after the last dose. In CMV-seropositive participants, mRNA-1647 demonstrated robust boosting of nAb and antigen-specific binding IgG levels compared with baseline. Cellular immune responses as measured by IFN-γ ELISpot were observed in all mRNA-1647 groups. Additionally, exploratory immunologic analyses indicate that mRNA-1647 evokes broad nAb responses as well as multifunctional T-cell responses [17].

Due to its substantial health impact, the Institute of Medicine (now National Academy of Medicine) Committee to Study Priorities for Vaccine Development placed CMV vaccine development in its most favorable category in 2000 [18]. Accordingly, several CMV vaccine candidates have been investigated using different approaches, including live-attenuated vaccine (Towne [19, 20]); replication-defective whole-virus vaccine (V160 [21–24]); adjuvanted gB subunit vaccine (gBMF59 [25–27]); recombinant modified vaccinia Ankara virus expressing pp65 and intermediate early (IE) antigens, IE1-exon4 and IE2-exon5 (Triplex [28–30]); and nonreplicating lymphocytic choriomeningitis virus vectors expressing gB and pp65 (HB-101 [31, 32]). Challenges associated with historical CMV vaccine development included limited duration of immune responses, failure to sufficiently reduce CMV infection in transplant recipients and healthy individuals, and difficulties in scaling vaccine production [14, 33]. As data from previous trials of CMV vaccine candidates collectively suggest that an effective CMV vaccine likely requires a multi-antigen approach [8], mRNA-1647 was designed utilizing mRNA vaccine technology to encode both the CMV antigens gB and pentamer. The mRNA vaccine platform affords the ability to efficiently design multi-antigenic vaccine candidates for rapid production and preclinical testing [14, 34], and the pentamer component of mRNA-1647 in particular is a novel example of the potential of this platform to leverage the naturally occurring host cell biology for in vivo production of a complex multimeric target antigen [14, 34].

To enable efficient development of CMV vaccines, a key focus is establishment of immunologic correlates of protection [35], which can serve as intermediate trial endpoints, thus allowing for more rapid identification of highly effective vaccines [35]. While a validated correlate of protection for CMV is not well established, the partial protection against CMV-related outcomes induced by natural CMV infection indicates that both humoral and cell-mediated immunity are important [35]. The presence of CMV-specific antibodies and T-cell responses have been correlated with reduced incidence and severity of congenital CMV infection [36–38]. Congenital CMV infection risk among infants born to CMV-seropositive mothers following nonprimary infection is considerably lower than those born to CMV-seronegative mothers who acquire primary CMV infection during pregnancy (3% vs 30%–70%, respectively), suggesting that preexisting maternal immunity is partly protective against fetal infection [8, 39]. Prior immunity is also an important factor in determining CMV infection risk in transplant recipients and reflects differences in underlying host immunobiology and influence of CMV strain-dependent immune responses. In a large cohort study, CMV infection risk in the hematopoietic cell transplant population was highest among CMV-seropositive recipients from CMV-seronegative donors (infection risk of 56%) and CMV-seropositive donors (53%), with lung transplant risk at 42% and 37% in CMV-seronegative and CMV-seropositive recipients, respectively, from CMV-seropositive donors [40]. Together, observations in pregnant and immunocompromised populations highlight that natural immunity alone is insufficient for protection against CMV. A CMV vaccine that induces robust humoral and cell-mediated immunity to prevent primary infection and reactivation of latent virus could therefore substantially impact the burden of CMV infection and disease. Furthermore, as acquired immunity appears to be insufficient to prevent reinfection, it will be important to evaluate the different components of the vaccine-elicited immune response and whether they translate to cross-strain protection over time [41, 42]. Such an evaluation could contribute to the currently limited body of work focusing on infection and immune response dynamics between CMV strains [41, 42].

Trial limitations include small sample sizes and inclusion of US adults 18–49 years of age, potentially preventing generalizability to other age groups and locations. Trial strengths are the randomized, observer-blind, placebo-controlled design and the inclusion of CMV-seronegative and CMV-seropositive participants, providing insight into serostatus-specific safety and immunogenicity.

Investigation of mRNA-1647 for the prevention of CMV infection is ongoing and the data generated in this phase 1 trial were vital to proceed with clinical development. Further insights into mRNA-1647 will be gained from the phase 2, randomized, observer-blind, placebo-controlled, dose-finding trial designed to evaluate the safety and immunogenicity of mRNA-1647 in healthy adults (NCT04232280). Additionally, a phase 3, randomized, observer-blind, placebo-controlled trial to evaluate the efficacy, safety, and immunogenicity of mRNA-1647 (100 µg) in healthy females is underway (NCT05085366).

## Supplementary Data

Supplementary materials are available at *The Journal of Infectious Diseases* online (http://jid.oxfordjournals.org/). Supplementary materials consist of data provided by the author that are published to benefit the reader. The posted materials are not copyedited. The contents of all supplementary data are the sole responsibility of the authors. Questions or messages regarding errors should be addressed to the author.

## Supplementary Material

jiae114_Supplementary_Data

## Notes

***Acknowledgments.*** We thank the participants in the trial and the members of the P101 trial team for their dedication and contributions to the trial, and the members of the safety monitoring committee for their hard work, support, and guidance of the trial. We also thank Kai Wu, PhD (Moderna, Inc), for his review and input on this manuscript. Medical writing and editorial assistance were provided by Kate Russin, PhD, of MEDiSTRAVA in accordance with Good Publication Practice guidelines, funded by Moderna, Inc, and under the direction of the authors.

***Author contributions*.** C. K., A. C., and L. P. were involved in the trial concept and design. Data collection was performed by C. F., D. B., M. S., H. S., C. K., J. L., and L. P. Analysis and interpretation of the data was undertaken by J. L., A. C., A. N., S. K., C. R., J. M., and L. P. All authors contributed to the drafting and critical review of this manuscript as well as approved the final draft.

***Data availability.*** Upon request, and subject to review, Moderna, Inc, will provide the data that support the findings of this trial. Subject to certain criteria, conditions, and exceptions, Moderna, Inc, may also provide access to the related individual anonymized participant data.

***Financial support.*** This work was supported by Moderna, Inc. Funding to pay the Open Access publication charges for this article was provided by Moderna, Inc.
