# Supplementary material for: Safety and Immunogenicity of a Messenger RNA–Based Cytomegalovirus Vaccine in Healthy Adults: Results From a Phase 1 Randomized Clinical Trial
Source: J Infect Dis. 2024 Mar 13;230(3):e668–78. doi: 10.1093/infdis/jiae114 (PMC11420795; doi:10.1093/infdis/jiae114)
Supplement: jiae114_Supplementary_Data [file jiae114_supplementary_data.docx]

# Supplementary Material

### Supplementary Methods

**Inclusion and Exclusion Criteria**

Each participant was required to meet the following criteria:

1. Male or female from 18 through 49 years of age.
2. Understood and agreed to comply with the trial procedures and provided written informed consent.
3. In the opinion of the Investigator, could and would comply with the protocol requirements.
4. Had a body mass index from 18 through 35 kg/m^2^.
5. Was in good general health.
6. Female participant of non-childbearing potential, defined as bilateral tubal ligation >1 year prior to screening, bilateral oophorectomy, or hysterectomy or menopause.
7. Female participant of childbearing potential could be enrolled in the trial if the participant: 1) had a negative pregnancy test at screening and on the day of injection, and 2) had practiced adequate contraception or abstained from all activities which could have led to pregnancy for 30 days prior to dose 1, and 3) had agreed to continue adequate contraception through 3 months following dose 3.
8. Male participant agreed to practice adequate contraception for 30 days prior to dose 1 and through 3 months following dose 3.

In brief, exclusion criteria included:

1. Acute or chronic clinically significant disease. Asymptomatic conditions or findings were not exclusionary if appropriately managed, stable, and unlikely to progress within the trial period.
2. Malignancy diagnosis within 10 years (excluding non-melanoma skin cancer).
3. If the participant was female and of childbearing potential, was pregnant or lactating, had not adhered to an adequate contraception method from at least 30 days before trial entry, or did not plan to do so for at least 3 months after dose 3.
4. Elevated liver function tests, with a toxicity score of grade ≥1 at screening.
5. Safety laboratory test results with a toxicity score of grade ≥1 at screening.
6. Received any investigational or non-registered product other than the trial vaccine within 30 days preceding dose 1 of trial vaccine or had plans for administration during the trial period.
7. Previously participated in an investigational trial involving lipid nanoparticles.
8. Positive test result for hepatitis B, hepatitis C, or human immunodeficiency virus.
9. Positive urine drug screen for any of the following non-prescription drugs of abuse: amphetamines, benzodiazepines, cocaine, methadone, opiates, and phencyclidine.
10. Chronic administration (defined as ≥14 days within 3 months before dose 1) of potentially hepatotoxic drugs or had other medical conditions that affected the liver (eg, alcohol abuse).
11. History of idiopathic urticaria.
12. Plans for administration or had been administered a vaccine not foreseen by the trial protocol within the period from 30 days before through 30 days after each trial injection, except for any licensed influenza vaccine administered ≥15 days before or after any trial injection.
13. Chronic administration of immunosuppressants or other immune-modifying drugs within 6 months before dose 1.
14. Administered immunoglobulins and/or blood products within the 3 months before the first trial injection or had plans for administration during the trial period.
15. Diagnosed or suspected immune-mediated disease or immunosuppressive condition.
16. History of hypersensitivity or severe reactions to previous vaccinations.
17. Bleeding disorder that was considered a contraindication to intramuscular injection or phlebotomy.
18. Acutely ill or febrile on presentation to the screening visit. Participants meeting this criterion may have been rescheduled for screening at a later date. Afebrile subjects with minor illnesses could be enrolled at the discretion of the Investigator.
19. Any medical, psychiatric, or occupational condition that, in the opinion of the Investigator, might have posed an additional risk to the participant due to participation in the trial or could interfere with the evaluation of the trial vaccines or the interpretation of trial results.
20. For dose escalation Phases A and B and the sentinel cohort of sentinel expansion Phase C, was seropositive for cytomegalovirus (CMV) at screening.
21. An immediate family member or a household member of trial personnel.
22. Donated >450 mL of whole blood or blood products within 30 days of dosing.
23. History of seizure disorder for which anticonvulsants were prescribed. The following were permitted: a reported history of seizure disorder but no seizures and no prescribed anticonvulsants within the last 5 years, and febrile seizures during childhood.

**Potentially Immune-Mediated Medical Conditions (Adverse Events of Special Interest)**

Gastrointestinal disorders: celiac disease, Crohn’s disease, ulcerative colitis, ulcerative proctitis

Liver disorders: autoimmune cholangitis, autoimmune hepatitis, primary biliary cirrhosis, primary sclerosing cholangitis

Metabolic diseases: Addison’s disease, autoimmune thyroiditis (including Hashimoto thyroiditis), diabetes mellitus type 1, Grave’s or Basedow’s disease

Musculoskeletal disorders: antisynthetase syndrome, dermatomyositis, juvenile chronic arthritis (including Still’s disease), mixed connective tissue disorder, polymyalgia rheumatic, polymyositis, psoriatic arthropathy, relapsing polychondritis, rheumatoid arthritis, scleroderma (including diffuse systemic form and CREST syndrome), spondyloarthritis (including ankylosing spondylitis, reactive arthritis [Reiter's Syndrome], and undifferentiated spondyloarthritis), systemic lupus erythematosus, systemic sclerosis

Neuroinflammatory disorders: acute disseminated encephalomyelitis (including site-specific variants [eg, noninfectious encephalitis, encephalomyelitis, myelitis, radiculomyelitis]), cranial nerve disorders (including paralyses/paresis [eg, Bell’s palsy]), Guillain-Barré syndrome (including Miller Fisher syndrome and other variants), immune-mediated peripheral neuropathies and plexopathies (including chronic inflammatory demyelinating polyneuropathy, multifocal motor neuropathy, and polyneuropathies associated with monoclonal gammopathy), multiple sclerosis, narcolepsy, optic neuritis, transverse myelitis, myasthenia gravis (including Eaton-Lambert syndrome)

Skin disorders: alopecia areata, autoimmune bullous skin diseases (including pemphigus, pemphigoid, and dermatitis herpetiformis), cutaneous lupus erythematosus, erythema nodosum, morphea, lichen planus, psoriasis, Sweet’s syndrome, vitiligo

Vasculitides: large vessel vasculitis (including giant cell arteritis such as Takayasu’s arteritis and temporal arteritis), medium-sized and/or small vessel vasculitis (including polyarteritis nodosa, Kawasaki’s disease, microscopic polyangiitis, Wegener’s granulomatosis, Churg-Strauss syndrome [allergic granulomatous angiitis], Buerger’s disease thromboangiitis obliterans, necrotizing vasculitis and anti-neutrophil cytoplasmic antibody [ANCA]-positive vasculitis [type unspecified], Henoch-Schönlein purpura, Bechet’s syndrome, and leukocytoclastic vasculitis)

Others: antiphospholipid syndrome, autoimmune hemolytic anemia, autoimmune glomerulonephritis (including IgA nephropathy, glomerulonephritis rapidly progressive, membranous glomerulonephritis, membranoproliferative glomerulonephritis, and mesangial proliferative glomerulonephritis), autoimmune myocarditis/cardiomyopathy, autoimmune thrombocytopenia, Goodpasture syndrome, idiopathic pulmonary fibrosis, pernicious anemia, Raynaud’s phenomenon, sarcoidosis, Sjögren’s syndrome, Stevens-Johnson syndrome, uveitis

**Immunogenicity Assays**

Cell-based assays were used to measure neutralizing antibody titers. Epithelial cell neutralization assays were performed with ARPE-19 cells using the human cytomegalovirus (HCMV) VR1814 strain. Fibroblast neutralization assays were performed with HEL299 cells cultured in 5% guinea pig complement using the HCMV AD169 strain. For each assay, a standard number of infectious units incubated with serial dilutions of sera was added to the cells which were incubated, ethanol/methanol-fixed, followed by incubation with a monoclonal antibody that targets the HCMV-IE1 protein, followed by secondary substrates that form a blue precipitate on IE1-positive cells. Images of all wells were acquired by a CTL ImmunoSpot® S6 UV Analyzer, equipped with BioSpot® software to quantitate the IE1-positive cells. The 50% neutralization titers were calculated according to the method described by Zielinska et al [1].

Pentamer-specific and glycoprotein B (gB)-specific binding immunoglobulin G (IgG) titers were measured using enzyme-linked immunosorbent assays (ELISAs) using recombinant CMV protein antigen and human serum or plasma samples. Wells were coated with CMV pentamer or gB antigens, and anti-human IgG antibody conjugated to peroxidase was added, followed by a substrate for color development. Optical density of samples was measured and used to calculate IgG titers [2].

Cell-mediated immune responses were determined by enzyme-linked immunospot (ELISpot) using the Human IFN-γ ELISpot^PRO^ Kit (Mabtech) and glycoprotein H (gH) and gB peptide pools (Genscript). Number of pentamer (gH)-specific and gB-specific T cells secreting interferon (IFN)-γ were reported as spot-forming cells (SFC)/10^6^ peripheral blood mononuclear cells (PBMCs).

**Data Analysis Sets**

Participant data reported herein were analyzed as follows: the randomized set, consisting of all enrolled participants who provided informed consent and were randomly assigned to treatment, regardless of the participant’s treatment status in the trial; the exposed set, consisting of all participants in the randomized set who received any trial injection; month 3, month 7, month 12, or month 18 antibody-mediated full analysis sets, consisting of all participants in the exposed set who provided evaluable serum samples before dose 1 (day 1) and ≥1 post-dose timepoint (month 3: month 1 or 3; month 7: month 6 or 7; month 12: month 12; month 18: month 18) and had results available for ≥1 antibody-mediated immunogenicity assay component at the respective timepoint; the full analysis set, consisting of all participants who were included in ≥1 of the month 3, month 7, month 12, or month 18 antibody-mediated full analysis sets; the per-protocol set, defined by timepoint and consisting of all evaluable participants in the full analysis set who met all eligibility criteria with no major protocol deviations, were administered trial dose according to the protocol, did not receive a concomitant medication or vaccine leading to exclusion from a per-protocol analysis, did not present with a medical condition that might have had an impact on the immunogenicity assessment, adhered to the injection schedule, complied with the timing of blood sampling at the corresponding timepoint, had immunogenicity results available for ≥1 assay component at the corresponding timepoint, and were appropriately characterized by CMV serostatus at screening and baseline; the cell-mediated immunogenicity set, consisting of all participants in the exposed set who provided evaluable PBMC samples at day 1, had at least 1 post-dose cell mediated result, and were appropriately characterized by CMV serostatus at screening and baseline; the solicited safety set, consisting of all participants in the exposed set who contributed any solicited local or systemic adverse reaction (AR); and the dose 1, dose 2, and dose 3 solicited safety sets, consisting of all participants in the solicited safety set who received doses 1, 2, and 3 and contributed any solicited local or systemic AR data from the time of injection through the following 6 days (dose 1: days 1 through 7; dose 2: month 2 through day 63; and dose 3: month 6 through day 175).

**References**

1. Zielinska E, Liu D, Wu HY, Quiroz J, Rappaport R, Yang DP. Development of an improved microneutralization assay for respiratory syncytial virus by automated plaque counting using imaging analysis. Virol J **2005**; 2:84.

2. Paila YD, Kapoor A, Mani S, et al. Validation of binding and neutralizing antibody assays for use in cytomegalovirus mRNA-1647 vaccine clinical trials. 8th International Congenital CMV Conference and the International CMV Workshop. Virtual, **2022**.

### Supplementary Results

**Treatment-Emergent Adverse Events Leading to Discontinuation From Study Vaccination**

All treatment-related AEs leading to discontinuation from study vaccination were non-serious events; these AEs were reported in 4 participants each who received mRNA-1647 30 µg and 180 µg; 1 participant each who received mRNA-1647 90 µg and 300 µg; and 1 participant who received mRNA-1443 40 µg.

- A participant who received mRNA-1647 30 μg experienced grade 3 events of headache and fatigue, both of which were assessed by the Investigator as treatment related.
- A participant who received mRNA-1647 30 μg experienced grade 2 events of injection site pain, pyrexia, and chills and grade 3 events of fatigue, arthralgia, myalgia, all of which were assessed by the Investigator as treatment related.
- A participant who received mRNA-1647 30 μg experienced a grade 1 event of pyrexia, grade 2 events of nausea, arthralgia, injection site pain, and headache, and grade 3 events of chills, myalgia, and fatigue, all of which were assessed by the Investigator as treatment related.
- A participant who received mRNA-1647 30 μg experienced a grade 2 medically attended AE (MAAE) of parvovirus B19 infection, which was assessed by the Investigator as not treatment related.
- A participant who received mRNA-1647 90 μg experienced a grade 3 MAAE of rhabdomyolysis on Day 2 after the receipt of dose 2. On Day 1 and Day 8 after the receipt of dose 3, the participant experienced a grade 3 non-serious event of blood pressure increased and a grade 3 event of hemoglobin decreased, respectively. The events of rhabdomyolysis, blood pressure increased, and hemoglobin decreased were all assessed by the Investigator as not treatment related.
- A participant who received mRNA-1647 180 μg experienced grade 3 events of pyrexia, back pain, chills, arthralgia, fatigue, and myalgia, all of which were assessed by the Investigator as treatment related.
- A participant who received mRNA-1647 180 μg experienced grade 3 events of chills, arthralgia, fatigue, myalgia, and pyrexia, all of which were assessed by the Investigator as treatment related.
- A participant who received mRNA-1647 180 μg experienced grade 2 events of chills, myalgia, fatigue, injection site pain, arthralgia, and headache, all of which were assessed by the Investigator as treatment related.
- A participant who received mRNA-1647 180 μg experienced grade 2 events of chills, fatigue, pyrexia, injection site pain, arthralgia, and myalgia, all of which were assessed by the Investigator as treatment related.
- A participant who received mRNA-1647 300 μg experienced a MAAE of grade 2 arthralgia, which was assessed by the Investigator as not treatment related.
- A participant who received mRNA-1443 40 μg experienced a grade 2 event of chills and a grade 3 MAAE of pyrexia, both of which were assessed by the Investigator as treatment related.

### Supplementary Table 1. Demographics of Phase A Participants^a^

|  |  | **mRNA-1647** | | |
| --- | --- | --- | --- | --- |
|  | **Placebo**  **(n=3)^b^** | **30 µg**  **(n=4)^b^** | **90 µg**  **(n=4)^b^** | **180 µg**  **(n=4)^b^** |
| **Sex, n (%)^c^** |  |  |  |  |
| Male | 1 (33.3) | 2 (50.0) | 1 (25.0) | 2 (50.0) |
| Female | 2 (66.7) | 2 (50.0) | 3 (75.0) | 2 (50.0) |
| **Age, mean (SD) years** | 37.3 (6.5) | 30.3 (5.9) | 34.3 (13.0) | 28.5 (11.2) |
| **Race, n (%)^c^** |  |  |  |  |
| White | 3 (100.0) | 3 (75.0) | 4 (100.0) | 4 (100.0) |
| Black/African American | 0 | 1 (25.0) | 0 | 0 |
| **Ethnicity, n (%)^c^** |  |  |  |  |
| Not Hispanic or Latino | 3 (100.0) | 4 (100.0) | 4 (100.0) | 4 (100.0) |
| **Mean weight, kg** | 92.53 (25.8) | 81.98 (18.0) | 83.73 (8.4) | 71.88 (12.4) |
| **Mean height, cm** | 178.23 (11.4) | 170.18 (13.6) | 165.75 (11.6) | 166.35 (3.4) |
| **Mean BMI, kg/m^2^** | 28.73 (5.0) | 28.03 (3.2) | 30.65 (4.0) | 26.03 (5.0) |

Abbreviations: BMI, body-mass index; CMV, cytomegalovirus.

^a^Phase A enrolled CMV-seronegative participants only. Data are from the exposed set, which consisted of all participants in the randomized set who received any trial injection.

^b^Number of participants exposed to placebo or mRNA-1647.

^c^Number of participants exposed to placebo or mRNA-1647 in category with non-missing data.

### Supplementary Table 2. Solicited Local and Systemic ARs in Phase A Participants^a^

|  | |  | | | **mRNA-1647** | | | | | | | | |
| --- | --- | --- | --- | --- | --- | --- | --- | --- | --- | --- | --- | --- | --- |
|  | | **Placebo** | | | **30 µg** | | | **90 µg** | | | **180 µg** | | |
|  | | **Dose 1**  **(n=3)^b^** | **Dose 2**  **(n=3)^b^** | **Dose 3**  **(n=3)^b^** | **Dose 1**  **(n=4)^b^** | **Dose 2**  **(n=4)^b^** | **Dose 3**  **(n=3)^b^** | **Dose 1**  **(n=4)^b^** | **Dose 2**  **(n=4)^b^** | **Dose 3**  **(n=4)^b^** | **Dose 1**  **(n=4)^b^** | **Dose 2**  **(n=4)^b^** | **Dose 3**  **(n=4)^b^** |
| **Local ARs,** n (%)^c^ | | | | | | | | | | | | | |
| Pain | |  |  |  |  |  |  |  |  |  |  |  |  |
|  | Grade 1 | 0 | 0 | 0 | 1 (25.0) | 1 (25.0) | 1 (33.3) | 3 (75.0) | 2 (50.0) | 2 (50.0) | 1 (25.0) | 1 (25.0) | 0 |
|  | Grade 2 | 0 | 0 | 0 | 0 | 0 | 0 | 0 | 0 | 0 | 3 (75.0) | 3 (75.0) | 4 (100.0) |
|  | Grade 3 | 0 | 0 | 0 | 0 | 0 | 0 | 0 | 0 | 0 | 0 | 0 | 0 |
| Redness (erythema) | |  |  |  |  |  |  |  |  |  |  |  |  |
|  | Grade 1 | 0 | 0 | 0 | 0 | 0 | 0 | 0 | 0 | 0 | 0 | 0 | 1 (25.0) |
|  | Grade 2 | 0 | 0 | 0 | 0 | 0 | 0 | 0 | 0 | 0 | 0 | 0 | 0 |
|  | Grade 3 | 0 | 0 | 0 | 0 | 0 | 0 | 0 | 0 | 0 | 0 | 0 | 0 |
| Hardness (swelling) | |  |  |  |  |  |  |  |  |  |  |  |  |
|  | Grade 1 | 0 | 0 | 0 | 0 | 0 | 1 (33.3) | 0 | 0 | 0 | 0 | 0 | 0 |
|  | Grade 2 | 0 | 0 | 0 | 0 | 0 | 0 | 0 | 0 | 0 | 0 | 0 | 0 |
|  | Grade 3 | 0 | 0 | 0 | 0 | 0 | 0 | 0 | 0 | 0 | 0 | 0 | 0 |
| **Systemic ARs,** n (%)^c^ | | | | | | | | | | | | | |
| Fever | |  |  |  |  |  |  |  |  |  |  |  |  |
|  | Grade 1 | 0 | 0 | 0 | 0 | 0 | 0 | 1 (25.0) | 0 | 0 | 0 | 0 | 0 |
|  | Grade 2 | 0 | 0 | 0 | 0 | 0 | 0 | 0 | 0 | 0 | 0 | 0 | 0 |
|  | Grade 3 | 0 | 0 | 0 | 0 | 0 | 0 | 0 | 0 | 0 | 0 | 0 | 0 |
| Headache | |  |  |  |  |  |  |  |  |  |  |  |  |
|  | Grade 1 | 1 (33.3) | 1 (33.3) | 1 (33.3) | 0 | 1 (25.0) | 1 (33.3) | 1 (25.0) | 0 | 1 (25.0) | 1 (25.0) | 1 (25.0) | 1 (25.0) |
|  | Grade 2 | 0 | 0 | 0 | 0 | 0 | 0 | 0 | 1 (25.0) | 1 (25.0) | 0 | 1 (25.0) | 2 (50.0) |
|  | Grade 3 | 0 | 0 | 0 | 0 | 0 | 0 | 0 | 0 | 0 | 0 | 0 | 0 |
| Fatigue | |  |  |  |  |  |  |  |  |  |  |  |  |
|  | Grade 1 | 0 | 0 | 1 (33.3) | 0 | 0 | 1 (33.3) | 1 (25.0) | 0 | 2 (50.0) | 1 (25.0) | 1 (25.0) | 3 (75.0) |
|  | Grade 2 | 0 | 0 | 0 | 0 | 0 | 0 | 0 | 1 (25.0) | 0 | 0 | 1 (25.0) | 1 (25.0) |
|  | Grade 3 | 0 | 0 | 0 | 0 | 0 | 0 | 0 | 0 | 0 | 0 | 0 | 0 |
| Myalgia | |  |  |  |  |  |  |  |  |  |  |  |  |
|  | Grade 1 | 0 | 0 | 0 | 0 | 0 | 1 (33.3) | 1 (25.0) | 1 (25.0) | 1 (25.0) | 0 | 1 (25.0) | 1 (25.0) |
|  | Grade 2 | 0 | 0 | 0 | 0 | 0 | 0 | 0 | 0 | 1 (25.0) | 1 (25.0) | 2 (50.0) | 1 (25.0) |
|  | Grade 3 | 0 | 0 | 0 | 0 | 0 | 0 | 0 | 0 | 0 | 0 | 0 | 0 |
| Arthralgia | |  |  |  |  |  |  |  |  |  |  |  |  |
|  | Grade 1 | 0 | 0 | 0 | 0 | 0 | 1 (33.3) | 1 (25.0) | 0 | 1 (25.0) | 0 | 1 (25.0) | 2 (50.0) |
|  | Grade 2 | 0 | 0 | 0 | 0 | 0 | 0 | 0 | 0 | 0 | 0 | 1 (25.0) | 0 |
|  | Grade 3 | 0 | 0 | 0 | 0 | 0 | 0 | 0 | 0 | 0 | 0 | 0 | 0 |
| Nausea | |  |  |  |  |  |  |  |  |  |  |  |  |
|  | Grade 1 | 0 | 0 | 0 | 0 | 0 | 0 | 1 (25.0) | 0 | 0 | 1 (25.0) | 1 (25.0) | 0 |
|  | Grade 2 | 0 | 0 | 0 | 0 | 0 | 0 | 0 | 1 (25.0) | 0 | 0 | 0 | 0 |
|  | Grade 3 | 0 | 0 | 0 | 0 | 0 | 0 | 0 | 0 | 0 | 0 | 0 | 0 |
| Chills | | NA | NA | NA | NA | NA | NA | NA | NA | NA | NA | NA | NA |

Abbreviations: AR, adverse reaction; CMV, cytomegalovirus.

^a^Phase A enrolled CMV-seronegative participants only. Data are from the dose 1, dose 2, and dose 3 solicited safety sets, which consisted of all participants in the solicited safety set who received dose 1, 2, or 3 and contributed any solicited local or systemic AR data from the time of injection through the following 6 days (dose 1: days 1 through 7; dose 2: month 2 through day 63; and dose 3: month 6 through day 175).

^b^Number of participants in the dose 1, dose 2, or dose 3 solicited safety set.

^c^Number of participants reporting the event.

### Supplementary Table 3. Summary of Unsolicited AEs in Each Phase^a^

|  | **CMV-Seronegative Participants** | | | | | | | | | |
| --- | --- | --- | --- | --- | --- | --- | --- | --- | --- | --- |
|  | **Phase A** | | | | **Phase B** | | | | **Phase C** | |
|  |  | **mRNA-1647** | | |  | **mRNA-1647** | | |  | **mRNA-1647** |
| n (%)^c^ | **Placebo**  **(n=3)** | **30 μg**  **(n=4)** | **90 μg**  **(n=4)** | **180 μg**  **(n=4)** | **Placebo**  **(n=14)^b^** | **30 µg**  **(n=17)^b^** | **90 µg**  **(n=15)^b^** | **180 µg**  **(n=16)^b^** | **Placebo**  **(n=3)^b^** | **300 µg**  **(n=15)^b^** |
| All unsolicited AEs | 1 (33.3) | 2 (50.0) | 4 (100.0) | 3 (75.0) | 6 (42.9) | 8 (47.1) | 7 (46.7) | 8 (50.0) | 3 (100.0) | 9 (60.0) |
| SAEs | 0 | 0 | 0 | 0 | 0 | 0 | 0 | 0 | 1 (33.3) | 0 |
| Fatal AEs | 0 | 0 | 0 | 0 | 0 | 0 | 0 | 0 | 0 | 0 |
| AEs leading to trial discontinuation | 0 | 0 | 0 | 0 | 0 | 0 | 0 | 0 | 0 | 0 |
| AEs grade 3 or 4 | 0 | 1 (25.0) | 1 (25.0) | 0 | 2 (14.3) | 1 (5.9) | 0 | 2 (12.5) | 2 (66.7) | 4 (26.7) |
| AESIs | 0 | 0 | 0 | 0 | 0 | 0 | 0 | 0 | 0 | 0 |
| MAAEs | 1 (33.3) | 2 (50.0) | 0 | 1 (50.0) | 4 (28.6) | 6 (35.3) | 3 (20.0) | 3 (18.8) | 3 (100.0) | 3 (20.0) |

|  | **CMV-Seropositive Participants** | | | | | |
| --- | --- | --- | --- | --- | --- | --- |
|  | **Phase B** | | | | **Phase C** | |
|  |  | **mRNA-1647** | | |  | **mRNA-1647** |
| n (%)^c^ | **Placebo**  **(n=16)^b^** | **30 µg**  **(n=13)^b^** | **90 µg**  **(n=15)^b^** | **180 µg**  **(n=13)^b^** | **Placebo**  **(n=3)^b^** | **300 µg**  **(n=14)^b^** |
| All unsolicited AEs | 10 (62.5) | 4 (30.8) | 8 (53.3) | 11 (84.6) | 0 | 8 (57.1) |
| SAEs | 0 | 0 | 0 | 0 | 0 | 1 (7.1) |
| Fatal AEs | 0 | 0 | 0 | 0 | 0 | 0 |
| AEs leading to trial discontinuation | 0 | 0 | 0 | 0 | 0 | 0 |
| AEs grade 3 or 4 | 2 (12.5) | 2 (15.4) | 2 (13.3) | 5 (38.5) | 0 | 6 (42.9) |
| AESIs | 0 | 0 | 0 | 0 | 0 | 0 |
| MAAEs | 4 (25.0) | 1 (7.7) | 2 (13.3) | 6 (46.2) | 0 | 3 (21.4) |

|  | **CMV-Seropositive and CMV-Seronegative**  **Phase B and Phase C Participants Combined** | | | | | |
| --- | --- | --- | --- | --- | --- | --- |
|  | **Phase B** | | | | **Phase C** | |
|  |  | **mRNA-1647** | | |  | **mRNA-1647** |
| n (%)^c^ | **Placebo**  **(n=30)^b^** | **30 µg**  **(n=30)^b^** | **90 µg**  **(n=30)^b^** | **180 µg**  **(n=29)^b^** | **Placebo**  **(n=6)^b^** | **300 µg**  **(n=29)^b^** |
| All unsolicited AEs | 16 (53.3) | 12 (40.0) | 15 (50.0) | 19 (65.5) | 3 (50.0) | 17 (58.6) |
| SAEs | 0 | 0 | 0 | 0 | 1 (16.7) | 1 (3.4) |
| Fatal AEs | 0 | 0 | 0 | 0 | 0 | 0 |
| AEs leading to trial discontinuation | 0 | 0 | 0 | 0 | 0 | 0 |
| AEs grade 3 or 4 | 4 (13.3) | 3 (10.0) | 2 (6.7) | 7 (24.1) | 2 (33.3) | 10 (34.5) |
| AESIs | 0 | 0 | 0 | 0 | 0 | 0 |
| MAAEs | 8 (26.7) | 7 (23.3) | 5 (16.7) | 9 (31.0) | 3 (50.0) | 6 (20.7) |

Abbreviations: AE, adverse event; AESI, adverse event of special interest; CMV, cytomegalovirus; MAAE, medically attended adverse event; SAE, serious adverse event.

^a^Data are from the exposed set, which consisted of all participants in the randomized set who received any trial injection. Data are unsolicited AEs regardless of relationship. Phase A enrolled CMV-seronegative participants only. Data are from the overall stage, which began at dose 1 on day 1 and continued through the final safety follow-up.

^b^Number of participants exposed to placebo or mRNA-1647.

^c^Number of participants exposed to placebo or mRNA-1647 reporting the event.

### Supplementary Table 4. Incidence of Unsolicited AEs Assessed as Treatment-Related^a^

| **Phase A** | | | | |
| --- | --- | --- | --- | --- |
|  | **CMV-Seronegative Participants** | | | |
|  |  | **mRNA-1647** | | |
|  | **Placebo**  **(n=3)^b^** | **30 µg**  **(n=4)^b^** | **90 µg**  **(n=4)^b^** | **180 µg (n=4)^b^** |
| n (%)^c^ | | | | |
| Chills | 0 | 1 (25.0) | 2 (50.0) | 2 (50.0) |
| Neutropenia | 0 | 0 | 1 (25.0) | 0 |
| Acute sinusitis | 0 | 0 | 0 | 0 |
| Back pain | 0 | 0 | 0 | 0 |
| Bronchitis | 0 | 0 | 0 | 0 |
| Headache | 0 | 0 | 0 | 0 |
| Injection site lymphadenopathy | 0 | 0 | 0 | 0 |
| Pyrexia | 0 | 0 | 0 | 0 |

| **Phase B^d^** | | | | | | | | | |
| --- | --- | --- | --- | --- | --- | --- | --- | --- | --- |
|  | **CMV-Seronegative Participants** | | | | **CMV-Seronegative Participants** | | | | |
|  |  | **mRNA-1647** | | |  | **mRNA-1647** | | | |
|  | **Placebo**  **(n=14)^b^** | **30 µg**  **(n=17)^b^** | **90 µg**  **(n=15)^b^** | **180 µg (n=16)^b^** | **Placebo**  **(n=16)^b^** | | **30 µg**  **(n=13)^b^** | **90 µg**  **(n=13)^b^** | **180 µg (n=15)^b^** |
| n (%)^c^ | | | | | | | | | |
| Fatigue | 0 | 1 (5.9) | 0 | 1 (6.3) | 0 | | 2 (15.4) | 0 | 4 (30.8) |
| Arthralgia | 0 | 1 (5.9) | 0 | 0 | 0 | | 1 (7.7) | 0 | 4 (30.8) |
| Chills | 0 | 1 (5.9) | 0 | 0 | 0 | | 1 (7.7) | 0 | 4 (30.8) |
| Injection site lymphadenopathy | 0 | 1 (5.9) | 0 | 1 (6.3) | 0 | | 0 | 1 (6.7) | 3 (23.1) |
| Myalgia | 0 | 1 (5.9) | 0 | 0 | 0 | | 1 (7.7) | 0 | 4 (30.8) |
| Pyrexia | 0 | 1 (5.9) | 0 | 0 | 0 | | 1 (7.7) | 0 | 4 (30.8) |

| **Phase C** | | | | |
| --- | --- | --- | --- | --- |
|  | **CMV-Seronegative Participants** | | **CMV-Seronegative Participants** | |
|  | **Placebo**  **(n=3)^b^** | **mRNA-1647**  **300 µg**  **(n=15)^b^** | **Placebo**  **(n=3)^b^** | **mRNA-1647**  **300 µg**  **(n=114)^b^** |
|  |  |  |  |  |
|  |  |  |  |  |
| n (%)^c^ |  |  |  |  |
| Injection site pain | 0 | 2 (13.3) | 0 | 2 (14.3) |
| Injection site erythema | 0 | 2 (13.3) | 0 | 0 |
| Arthralgia | 0 | 1 (6.7) | 0 | 0 |
| Dizziness | 0 | 1 (6.7) | 0 | 0 |
| Gastroenteritis | 0 | 0 | 0 | 1 (7.1) |
| Hemoglobin decreased | 0 | 0 | 0 | 1 (7.1) |
| Injection site bruising | 0 | 1 (6.7) | 0 | 0 |
| Injection site induration | 0 | 1 (6.7) | 0 | 0 |
| Injection site pruritis | 0 | 0 | 0 | 1 (7.1) |
| Migraine | 0 | 0 | 0 | 1 (7.1) |
| Night sweats | 0 | 1 (6.7) | 0 | 0 |
| Pyrexia | 0 | 0 | 0 | 1 (7.1) |

Abbreviations: AE, adverse event; CMV, cytomegalovirus.

^a^Data are from the exposed set, which consisted of all participants in the randomized set who received any trial injection. Phase A enrolled CMV-seronegative participants only. The timeframe for data collection was dose 1 on day 1 through the final safety follow-up.

^b^Number of exposed participants.

^c^Number of exposed participants reporting the event.

### Supplementary Table 5. Demographics by CMV Serostatus^a^

|  | **CMV-Seronegative Participants** | | | | | | | | **CMV-Seropositive Participants** | | | | | | | |
| --- | --- | --- | --- | --- | --- | --- | --- | --- | --- | --- | --- | --- | --- | --- | --- | --- |
|  |  | |  | **mRNA-1647** | | | | |  | | | | **mRNA-1647** | | | |
|  | **Placebo Phase B**  **(n=14)^b^** | **Placebo, Phase C**  **(n=3)^b^** | | | **30 µg**  **(n=17)^b^** | **90 µg**  **(n=15)^b^** | **180 µg**  **(n=16)^b^** | **300 µg (n=15)^b^** | | **Placebo Phase B**  **(n=16) ^b^** | **Placebo Phase C**  **(n=3)^b^** | **30 µg (n=13)^b^** | | **90 µg (n=15)^b^** | **180 µg**  **(n=13)^b^** | **300 µg**  **(n=14)^b^** |
| **Sex, n (%)^c^** |  |  | | |  |  |  |  | |  |  |  | |  |  |  |
| Male | 9 (64.3) | 2 (66.7) | | | 5 (29.4) | 8 (53.3) | 5 (31.3) | 6 (40.0) | | 2 (12.5) | 2 (66.7) | 5 (38.5) | | 4 (26.7) | 5 (38.5) | 7 (50.0) |
| Female | 5 (35.7) | 1 (33.3) | | | 12 (70.6) | 7 (46.7) | 11 (68.8) | 9 (60.0) | | 14 (87.5) | 1 (33.3) | 8 (61.5) | | 11 (73.3) | 8 (61.5) | 7 (50.0) |
| **Mean age, y (SD)** | 33.5 (9.5) | 44.0 (3.0) | | | 33.6 (8.2) | 34.7 (8.8) | 32.0 (9.4) | 34.0 (8.6) | | 31.6 (7.8) | 41.0 (8.9) | 39.2 (6.7) | | 36.1 (8.5) | 36.4 (10.7) | 32.5 (9.0) |
| **Race, n (%)^c^** |  |  | | |  |  |  |  | |  |  |  | |  |  |  |
| White | 11 (78.6) | 3 (100) | | | 17 (100) | 13 (86.7) | 14 (87.5) | 12 (80.0) | | 13 (81.3) | 2 (66.7) | 11 (84.6) | | 11 (73.3) | 12 (92.3) | 12 (85.7) |
| Black/  African American | 2 (14.3) | 0 | | | 0 | 2 (13.3) | 1 (6.3) | 2 (13.3) | | 3 (18.8) | 1 (33.3) | 2 (15.4) | | 4 (26.7) | 1 (7.7) | 2 (14.3) |
| Asian | 0 | 0 | | | 0 | 0 | 1 (6.3) | 0 | | 0 | 0 | 0 | | 0 | 0 | 0 |
| American Indian or Alaska Native | 1 (7.1) | 0 | | | 0 | 0 | 0 | 0 | | 0 | 0 | 0 | | 0 | 0 | 0 |
| Not Reported | 0 | 0 | | | 0 | 0 | 0 | 1 (6.7) | | 0 | 0 | 0 | | 0 | 0 | 0 |
| **Ethnicity, n (%)^c^** | | | | | | | | | | | | | | | | |
| Not Hispanic or Latino | 12 (85.7) | 3 (100) | | | 17 (100) | 15 (100) | 16 (100) | 12 (80.0) | | 15 (93.8) | 2 (66.7) | 12 (92.3) | | 15 (100) | 13 (100) | 8 (57.1) |
| Hispanic or Latino | 2 (14.3) | 0 | | | 0 | 0 | 0 | 3 (20.0) | | 1 (6.3) | 1 (33.3) | 1 (7.7) | | 0 | 0 | 6 (42.9) |
| **CMV serostatus at screening, n (%)^c^** | | | | | | | | | | | | | | | | |
| Negative | 14 (100) | 3 (100) | | | 17 (100) | 15 (100) | 16 (100) | 15 (100) | | 2 (12.5) | 0 | 0 | | 2 (13.3) | 0 | 0 |
| Positive | 0 | 0 | | | 0 | 0 | 0 | 0 | | 14 (87.5) | 3 (100) | 13 (100) | | 13 (86.7) | 13 (100) | 14 (100) |
| **CMV serostatus at baseline, n (%)^c,d^** | | | | | | | | | | | | | | | | |
| Negative | 14 (100) | 3(100) | | | 17 (100) | 15 (100) | 16 (100) | 15 (100) | | 0 | 0 | 0 | | 0 | 0 | 0 |
| Positive | 0 | 0 | | | 0 | 0 | 0 | 0 | | 16 (100) | 3 (100) | 13 (100) | | 15 (100) | 13 (100) | 14 (100) |

Abbreviations: AE, adverse event; CMV, cytomegalovirus.

^a^Data are from the exposed set, which consisted of all participants in the randomized set who received any trial injection. The timeframe for data collection was dose 1 on day 1 through the final safety follow-up.

^b^Number of exposed participants.

^c^Number of exposed participants reporting the event.

### Supplementary Table 6. Solicited Local and Systemic ARs in Phases B and C in CMV-Seronegative Participants^a^

|  | **Phase B** | | | | | | | | | | | | | | **Phase C** | | | | | | |
| --- | --- | --- | --- | --- | --- | --- | --- | --- | --- | --- | --- | --- | --- | --- | --- | --- | --- | --- | --- | --- | --- |
|  | **Placebo** | | | | **mRNA-1647** | | | | | | | | | | **Placebo** | | | **mRNA-1647** | | | |
|  |  |  |  |  | **30 µg** | | | | **90 µg** | | | **180 µg** | | |  |  |  | **300 µg** | | | |
|  | **Dose 1** | **Dose 2** | **Dose 3** | **Dose 1** | | **Dose 2** | **Dose 3** | **Dose 1** | | **Dose 2** | **Dose 3** | **Dose 1** | **Dose 2** | **Dose 3** | **Dose 1** | **Dose 2** | **Dose 3** | | **Dose 1** | **Dose 2** | **Dose 3** |
| n**^b^** | **14** | **12** | **11** | **17** | | **15** | **11** | **15** | | **14** | **13** | **16** | **15** | **12** | **3** | **3** | **3** | | **14** | **12** | **14** |
| Local ARs, n (%)^b^ | | | | | | | | | | | | | | | | | | | | | |
| **Any local AR** | 2 (14.3) | 0 | 1 (9.1) | 13 (76.5) | | 11 (73.3) | 8 (72.7) | 11 (73.3) | | 11 (78.6) | 7 (53.8) | 16 (100.0) | 12 (80.0) | 9 (75.0) | 0 | 0 | 1 (33.3) | | 13 (92.9) | 11 (91.7) | 9 (90.0) |
| **Pain** |  |  |  |  | |  |  |  | |  |  |  |  |  |  |  |  | |  |  |  |
| Grade 1 | 2 (14.3) | 0 | 1 (9.1) | 11 (64.7) | | 7 (46.7) | 5 (45.5) | 7 (46.7) | | 7 (50.0) | 5 (38.5) | 8 (50.0) | 4 (26.7) | 2 (16.7) | 0 | 0 | 1 (33.3) | | 6 (42.9) | 4 (33.3) | 6 (66.7) |
| Grade 2 | 0 | 0 | 0 | 2 (11.8) | | 4 (26.7) | 3 (27.3) | 4 (26.7) | | 2 (14.3) | 1 (7.7) | 7 (43.8) | 6 (40.0) | 6 (50.0) | 0 | 0 | 0 | | 6 (42.9) | 5 (41.7) | 1 (11.1) |
| Grade 3 | 0 | 0 | 0 | 0 | | 0 | 0 | 0 | | 2 (14.3) | 1 (7.7) | 1 (6.3) | 2 (13.3) | 1 (8.3) | 0 | 0 | 0 | | 1 (7.1) | 2 (16.7) | 2 (22.2) |
| **Redness (erythema)** |  |  |  |  | |  |  |  | |  |  |  |  |  |  |  |  | |  |  |  |
| Grade 1 | 0 | 0 | 0 | 0 | | 0 | 0 | 0 | | 0 | 0 | 1 (6.3) | 2 (14.3) | 1 (8.3) | 0 | 0 | 0 | | 1 (10.0) | 0 | 0 |
| Grade 2 | 0 | 0 | 0 | 0 | | 0 | 0 | 0 | | 0 | 2 (15.4) | 0 | 1 (7.1) | 0 | 0 | 0 | 0 | | 0 | 1 (11.1) | 0 |
| Grade 3 | 0 | 0 | 0 | 0 | | 0 | 0 | 0 | | 0 | 0 | 0 | 0 | 0 | 0 | 0 | 0 | | 0 | 1 (11.1) | 1 (12.5) |
| **Hardness (swelling)** |  |  |  |  | |  |  |  | |  |  |  |  |  |  |  |  | |  |  |  |
| Grade 1 | 0 | 0 | 0 | 0 | | 0 | 0 | 0 | | 0 | 0 | 0 | 0 | 1 (8.3) | 0 | 0 | 0 | | 1 (10.0) | 0 | 0 |
| Grade 2 | 0 | 0 | 0 | 0 | | 0 | 0 | 0 | | 0 | 1 (7.7) | 0 | 0 | 0 | 0 | 0 | 0 | | 0 | 1 (12.5) | 0 |
| Grade 3 | 0 | 0 | 0 | 0 | | 0 | 0 | 0 | | 0 | 0 | 0 | 0 | 0 | 0 | 0 | 0 | | 0 | 1 (12.5) | 1 (12.5) |
| Systemic ARs, n (%)^b^ | | | | | | | | | | | | | | | | | | | | | |
| **Any systemic AR** | 5 (35.7) | 4 (33.3) | 1 (9.1) | 4 (23.5) | | 7 (46.7) | 5 (45.5) | 5 (33.3) | | 9 (64.3) | 8 (61.5) | 9 (56.3) | 13 (86.7) | 9 (75.0) | 0 | 1 (33.3) | 1 (33.3) | | 11 (78.6) | 11 (91.7) | 9 (90.0) |
| **Fever** |  |  |  |  | |  |  |  | |  |  |  |  |  |  |  |  | |  |  |  |
| Grade 1 | 0 | 0 | 0 | 0 | | 3 (20.0) | 0 | 0 | | 1 (7.1) | 1 (7.7) | 0 | 2 (13.3) | 2 (16.7) | 0 | 1 (33.3) | 0 | | 2 (14.3) | 2 (16.7) | 1 (10.0) |
| Grade 2 | 0 | 0 | 0 | 0 | | 0 | 1 (9.1) | 0 | | 1 (7.1) | 0 | 1 (6.3) | 3 (20.0) | 1 (8.3) | 0 | 0 | 0 | | 0 | 3 (25.0) | 1 (10.0) |
| Grade 3 | 0 | 0 | 0 | 0 | | 0 | 0 | 0 | | 1 (7.1) | 0 | 0 | 0 | 0 | 0 | 0 | 0 | | 0 | 1 (8.3) | 1 (10.0) |
| **Headache** |  |  |  |  | |  |  |  | |  |  |  |  |  |  |  |  | |  |  |  |
| Grade 1 | 2 (14.3) | 3 (25.0) | 1 (9.1) | 0 | | 1 (6.7) | 2 (18.2) | 4 (26.7) | | 4 (28.6) | 4 (30.8) | 3 (18.8) | 3 (20.0) | 3 (25.0) | 0 | 0 | 0 | | 2 (14.3) | 5 (41.7) | 4 (44.4) |
| Grade 2 | 0 | 0 | 0 | 1 (5.9) | | 4 (26.7) | 2 (18.2) | 0 | | 4 (28.6) | 0 | 0 | 4 (26.7) | 1 (8.3) | 0 | 0 | 0 | | 4 (28.6) | 4 (33.3) | 2 (22.2) |
| Grade 3 | 0 | 0 | 0 | 0 | | 0 | 0 | 0 | | 0 | 0 | 0 | 2 (13.3) | 2 (16.7) | 0 | 0 | 0 | | 0 | 0 | 1 (11.1) |
| **Fatigue** |  |  |  |  | |  |  |  | |  |  |  |  |  |  |  |  | |  |  |  |
| Grade 1 | 1 (7.1) | 2 (16.7) | 1 (9.1) | 2 (11.8) | | 2 (13.3) | 3 (27.3) | 2 (13.3) | | 3 (21.4) | 2 (15.4) | 5 (31.3) | 2 (13.3) | 2 (16.7) | 0 | 0 | 0 | | 5 (35.7) | 5 (41.7) | 2 (20.0) |
| Grade 2 | 0 | 0 | 0 | 0 | | 2 (13.3) | 1 (9.1) | 1 (6.7) | | 2 (14.3) | 2 (15.4) | 1 (6.3) | 8 (53.3) | 3 (25.0) | 0 | 0 | 0 | | 3 (21.4) | 4 (33.3) | 4 (40.0) |
| Grade 3 | 0 | 0 | 0 | 0 | | 1 (6.7) | 0 | 0 | | 1 (7.1) | 1 (7.7) | 0 | 1 (6.7) | 2 (16.7) | 0 | 0 | 0 | | 1 (7.1) | 0 | 1 (10.0) |
| **Myalgia** |  |  |  |  | |  |  |  | |  |  |  |  |  |  |  |  | |  |  |  |
| Grade 1 | 0 | 1 (8.3) | 0 | 0 | | 1 (6.7) | 2 (18.2) | 1 (6.7) | | 2 (14.3) | 3 (23.1) | 3 (18.8) | 3 (20.0) | 2 (16.7) | 0 | 0 | 0 | | 1 (7.1) | 3 (27.3) | 5 (50.0) |
| Grade 2 | 0 | 0 | 0 | 0 | | 3 (20.0) | 1 (9.1) | 1 (6.7) | | 1 (7.1) | 2 (15.4) | 1 (6.3) | 6 (40.0) | 2 (16.7) | 0 | 1 (33.3) | 0 | | 4 (28.6) | 3 (27.3) | 1 (10.0) |
| Grade 3 | 0 | 0 | 0 | 0 | | 1 (6.7) | 0 | 0 | | 3 (21.4) | 1 (7.7) | 1 (6.3) | 2 (13.3) | 2 (16.7) | 0 | 0 | 0 | | 0 | 1 (9.1) | 1 (10.0) |
| **Arthralgia** |  |  |  |  | |  |  |  | |  |  |  |  |  |  |  |  | |  |  |  |
| Grade 1 | 0 | 0 | 0 | 2 (11.8) | | 1 (6.7) | 2 (18.2) | 2 (13.3) | | 3 (21.4) | 4 (30.8) | 1 (6.3) | 3 (20.0) | 2 (16.7) | 0 | 0 | 0 | | 3 (21.4) | 1 (9.1) | 3 (30.0) |
| Grade 2 | 0 | 0 | 0 | 0 | | 3 (20.0) | 1 (9.1) | 0 | | 2 (14.3) | 1 (7.7) | 0 | 4 (26.7) | 4 (33.3) | 0 | 1 (33.3) | 0 | | 0 | 2 (18.2) | 0 |
| Grade 3 | 0 | 0 | 0 | 0 | | 0 | 0 | 0 | | 0 | 0 | 0 | 2 (13.3) | 0 | 0 | 0 | 0 | | 0 | 1 (9.1) | 1 (10.0) |
| **Nausea** |  |  |  |  | |  |  |  | |  |  |  |  |  |  |  |  | |  |  |  |
| Grade 1 | 0 | 1 (8.3) | 0 | 0 | | 3 (20.0) | 2 (18.2) | 2 (13.3) | | 2 (14.3) | 3 (23.1) | 3 (18.8) | 4 (26.7) | 2 (16.7) | 0 | 0 | 1 (33.3) | | 4 (28.6) | 2 (18.2) | 2 (20.0) |
| Grade 2 | 1 (7.1) | 0 | 0 | 0 | | 1 (6.7) | 0 | 1 (6.7) | | 2 (14.3) | 0 | 1 (6.3) | 2 (13.3) | 2 (16.7) | 0 | 0 | 0 | | 0 | 1 (9.1) | 1 (10.0) |
| Grade 3 | 0 | 0 | 0 | 0 | | 0 | 0 | 0 | | 0 | 0 | 0 | 1 (6.7) | 0 | 0 | 0 | 0 | | 1 (7.1) | 1 (9.1) | 0 |
| **Chills** |  |  |  |  | |  |  |  | |  |  |  |  |  |  |  |  | |  |  |  |
| Grade 1 | 1 (7.1) | 1 (8.3) | 0 | 1 (5.9) | | 1 (6.7) | 2 (18.2) | 1 (6.7) | | 2 (14.3) | 3 (23.1) | 2 (12.5) | 3 (20.0) | 1 (8.3) | 0 | 0 | 0 | | 1 (7.1) | 3 (27.3) | 1 (10.0) |
| Grade 2 | 0 | 0 | 0 | 0 | | 1 (6.7) | 0 | 0 | | 3 (21.4) | 2 (15.4) | 2 (12.5) | 5 (33.3) | 2 (16.7) | 0 | 1 (33.3) | 0 | | 5 (35.7) | 4 (36.4) | 3 (30.0) |
| Grade 3 | 0 | 0 | 0 | 0 | | 1 (6.7) | 0 | 0 | | 1 (7.1) | 0 | 0 | 3 (20.0) | 1 (8.3) | 0 | 0 | 0 | | 0 | 1 (9.1) | 1 (10.0) |

Data are from the dose 1, dose 2, and dose 3 solicited safety sets, which consisted of all participants in the solicited safety set who received dose 1, 2, or 3 and contributed any solicited local or systemic AR data from the time of injection through the following 6 days (dose 1: days 1 through 7; dose 2: month 2 through day 63; and dose 3: month 6 through day 175).

^a^Number of participants in the dose 1, dose 2, or dose 3 solicited safety set.

^b^Number of participants reporting the event.

### Supplementary Table 7. Solicited Local and Systemic ARs in Phases B and C in CMV-Seropositive Participants^a^

|  | **Phase B** | | | | | | | | | | | | | | **Phase C** | | | | | | |
| --- | --- | --- | --- | --- | --- | --- | --- | --- | --- | --- | --- | --- | --- | --- | --- | --- | --- | --- | --- | --- | --- |
|  | **Placebo** | | | | **mRNA-1647** | | | | | | | | | | **Placebo** | | | **mRNA-1647** | | | |
|  |  |  |  |  | **30 µg** | | | **90 µg** | | | | **180 µg** | | |  |  |  | **300 µg** | | | |
|  | **Dose 1** | **Dose 2** | **Dose 3** | **Dose 1** | | **Dose 2** | **Dose 3** | **Dose 1** | **Dose 2** | **Dose 3** | **Dose 1** | | **Dose 2** | **Dose 3** | **Dose 1** | **Dose 2** | **Dose 3** | | **Dose 1** | **Dose 2** | **Dose 3** |
| n**^a^** | **15** | **14** | **13** | **13** | | **12** | **11** | **15** | **13** | **10** | **13** | | **11** | **6** | **3** | **3** | **3** | | **14** | **12** | **14** |
| Local ARs, n (%)^b^ | | | | | | | | | | | | | | | | | | | | | |
| **Any local AR** | 1 (6.7) | 0 | 0 | 10 (76.9) | | 9 (75.0) | 8 (72.7) | 12 (80.0) | 10 (76.9) | 6 (60.0) | 13 (100.0) | | 10 (90.9) | 6 (100.0) | 0 | 0 | 0 | | 11 (91.7) | 9 (100.0) | 8 (100.0) |
| **Pain** |  |  |  |  | |  |  |  |  |  |  | |  |  |  |  |  | |  |  |  |
| Grade 1 | 1 (6.7) | 0 | 0 | 7 (53.8) | | 3 (25.0) | 7 (63.6) | 7 (46.7) | 6 (46.2) | 3 (30.0) | 4 (30.8) | | 2 (18.2) | 4 (66.7) | 0 | 0 | 0 | | 3 (27.3) | 1 (11.1) | 2 (25.0) |
| Grade 2 | 0 | 0 | 0 | 2 (15.4) | | 5 (41.7) | 1 (9.1) | 5 (33.3) | 4 (30.8) | 3 (30.0) | 6 (46.2) | | 5 (45.5) | 2 (33.3) | 0 | 0 | 0 | | 7 (63.6) | 5 (55.6) | 3 (37.5) |
| Grade 3 | 0 | 0 | 0 | 1 (7.7) | | 1 (8.3) | 0 | 0 | 0 | 0 | 3 (23.1) | | 3 (27.3) | 0 | 0 | 0 | 0 | | 1 (9.1) | 3 (33.3) | 3 (37.5) |
| **Redness (erythema)** |  |  |  |  | |  |  |  |  |  |  | |  |  |  |  |  | |  |  |  |
| Grade 1 | 0 | 0 | 0 | 0 | | 0 | 0 | 0 | 0 | 2 (20.0) | 0 | | 0 | 1 (16.7) | 0 | 0 | 0 | | 0 | 0 | 1 (12.5) |
| Grade 2 | 0 | 0 | 0 | 0 | | 0 | 0 | 0 | 0 | 0 | 2 (15.4) | | 1 (9.1) | 0 | 0 | 0 | 0 | | 0 | 0 | 0 |
| Grade 3 | 0 | 0 | 0 | 0 | | 0 | 0 | 0 | 0 | 0 | 0 | | 1 (9.1) | 0 | 0 | 0 | 0 | | 0 | 0 | 0 |
| **Hardness (swelling)** |  |  |  |  | |  |  |  |  |  |  | |  |  |  |  |  | |  |  |  |
| Grade 1 | 0 | 0 | 0 | 1 (7.7) | | 0 | 0 | 1 (6.7) | 0 | 1 (10.0) | 0 | | 1 (9.1) | 0 | 0 | 0 | 0 | | 1 (8.3) | 1 (14.3) | 2 (25.0) |
| Grade 2 | 0 | 0 | 0 | 0 | | 0 | 0 | 0 | 0 | 0 | 1 (7.7) | | 0 | 0 | 0 | 0 | 0 | | 0 | 0 | 0 |
| Grade 3 | 0 | 0 | 0 | 0 | | 0 | 0 | 0 | 0 | 0 | 0 | | 0 | 0 | 0 | 0 | 0 | | 0 | 0 | 0 |
| Systemic ARs, n (%)^b^ | | | | | | | | | | | | | | | | | | | | | |
| **Any systemic AR** | 5 (33.3) | 2 (14.3) | 4 (30.8) | 6 (46.2) | | 7 (58.3) | 5 (45.5) | 11 (73.3) | 9 (69.2) | 7 (70.0) | 12 (92.3) | | 11 (100.0) | 6 (100.0) | 1 (33.3) | 0 | 0 | | 13 (92.9) | 9 (100.0) | 8 (100.0) |
| **Fever** |  |  |  |  | |  |  |  |  |  |  | |  |  |  |  |  | |  |  |  |
| Grade 1 | 0 | 0 | 0 | 1 (7.7) | | 1 (8.3) | 0 | 2 (13.3) | 2 (15.4) | 2 (20.0) | 5 (38.5) | | 2 (18.2) | 1 (16.7) | 0 | 0 | 0 | | 2 (14.3) | 2 (22.2) | 2 (25.0) |
| Grade 2 | 0 | 0 | 0 | 0 | | 1 (8.3) | 1 (9.1) | 1 (6.7) | 1 (7.7) | 1 (10.0) | 1 (7.7) | | 2 (18.2) | 1 (16.7) | 0 | 0 | 0 | | 3 (21.4) | 1 (11.1) | 2 (25.0) |
| Grade 3 | 0 | 0 | 0 | 0 | | 0 | 0 | 1 (6.7) | 3 (23.1) | 1 (10.0) | 2 (15.4) | | 2 (18.2) | 1 (16.7) | 0 | 0 | 0 | | 2 (14.3) | 3 (33.3) | 2 (25.0) |
| **Headache** |  |  |  |  | |  |  |  |  |  |  | |  |  |  |  |  | |  |  |  |
| Grade 1 | 3 (20.0) | 2 (14.3) | 2 (15.4) | 2 (15.4) | | 0 | 3 (27.3) | 5 (33.3) | 3 (23.1) | 2 (20.0) | 3 (23.1) | | 3 (27.3) | 3 (50.0) | 1 (33.3) | 0 | 0 | | 5 (35.7) | 1 (11.1) | 3 (37.5) |
| Grade 2 | 0 | 0 | 1 (7.7) | 1 (7.7) | | 3 (25.0) | 1 (9.1) | 1 (6.7) | 3 (23.1) | 4 (40.0) | 6 (46.2) | | 4 (36.4) | 1 (16.7) | 0 | 0 | 0 | | 2 (14.3) | 5 (55.6) | 1 (12.5) |
| Grade 3 | 0 | 0 | 0 | 1 (7.7) | | 1 (8.3) | 1 (9.1) | 1 (6.7) | 0 | 0 | 0 | | 2 (18.2) | 1 (16.7) | 0 | 0 | 0 | | 3 (21.4) | 2 (22.2) | 2 (25.0) |
| **Fatigue** |  |  |  |  | |  |  |  |  |  |  | |  |  |  |  |  | |  |  |  |
| Grade 1 | 3 (20.0) | 1 (7.1) | 1 (7.7) | 0 | | 3 (25.0) | 3 (27.3) | 3 (20.0) | 2 (15.4) | 2 (20.0) | 2 (15.4) | | 0 | 1 (16.7) | 0 | 0 | 0 | | 2 (14.3) | 2 (22.2) | 2 (25.0) |
| Grade 2 | 0 | 0 | 0 | 1 (7.7) | | 1 (8.3) | 2 (18.2) | 1 (6.7) | 6 (46.2) | 5 (50.0) | 5 (38.5) | | 5 (45.5) | 2 (33.3) | 1 (33.3) | 0 | 0 | | 2 (14.3) | 3 (33.3) | 1 (12.5) |
| Grade 3 | 0 | 0 | 0 | 2 (15.4) | | 3 (25.0) | 0 | 1 (6.7) | 1 (7.7) | 0 | 2 (15.4) | | 3 (27.3) | 1 (16.7) | 0 | 0 | 0 | | 2 (14.3) | 2 (22.2) | 2 (25.0) |
| **Myalgia** |  |  |  |  | |  |  |  |  |  |  | |  |  |  |  |  | |  |  |  |
| Grade 1 | 1 (6.7) | 0 | 0 | 2 (15.4) | | 0 | 2 (18.2) | 5 (33.3) | 3 (23.1) | 2 (20.0) | 3 (23.1) | | 0 | 2 (33.3) | 0 | 0 | 0 | | 2 (14.3) | 1 (11.1) | 0 |
| Grade 2 | 0 | 0 | 0 | 1 (7.7) | | 1 (8.3) | 2 (18.2) | 1 (6.7) | 5 (38.5) | 4 (40.0) | 5 (38.5) | | 5 (45.5) | 1 (16.7) | 0 | 0 | 0 | | 4 (28.6) | 5 (55.6) | 3 (37.5) |
| Grade 3 | 0 | 0 | 0 | 0 | | 4 (33.3) | 1 (9.1) | 2 (13.3) | 1 (7.7) | 0 | 1 (7.7) | | 2 (18.2) | 0 | 0 | 0 | 0 | | 2 (14.3) | 1 (11.1) | 2 (25.0) |
| **Arthralgia** |  |  |  |  | |  |  |  |  |  |  | |  |  |  |  |  | |  |  |  |
| Grade 1 | 0 | 0 | 0 | 2 (15.4) | | 1 (8.3) | 3 (27.3) | 2 (13.3) | 2 (15.4) | 3 (30.0) | 1 (7.7) | | 0 | 2 (33.3) | 0 | 0 | 0 | | 2 (14.3) | 4 (44.4) | 2 (25.0) |
| Grade 2 | 0 | 0 | 0 | 1 (7.7) | | 1 (8.3) | 1 (9.1) | 1 (6.7) | 4 (30.8) | 1 (10.0) | 1 (7.7) | | 3 (27.3) | 0 | 0 | 0 | 0 | | 5 (35.7) | 3 (33.3) | 2 (25.0) |
| Grade 3 | 0 | 0 | 0 | 0 | | 3 (25.0) | 0 | 0 | 0 | 0 | 1 (7.7) | | 2 (18.2) | 0 | 0 | 0 | 0 | | 0 | 1 (11.1) | 2 (25.0) |
| **Nausea** |  |  |  |  | |  |  |  |  |  |  | |  |  |  |  |  | |  |  |  |
| Grade 1 | 2 (13.3) | 0 | 1 (7.7) | 0 | | 0 | 2 (18.2) | 2 (13.3) | 2 (15.4) | 3 (30.0) | 2 (15.4) | | 4 (36.4) | 0 | 1 (33.3) | 0 | 0 | | 4 (28.6) | 4 (44.4) | 2 (25.0) |
| Grade 2 | 0 | 0 | 0 | 1 (7.7) | | 1 (8.3) | 1 (9.1) | 1 (6.7) | 4 (30.8) | 2 (20.0) | 2 (15.4) | | 1 (9.1) | 1 (16.7) | 0 | 0 | 0 | | 2 (14.3) | 1 (11.1) | 0 |
| Grade 3 | 0 | 0 | 0 | 0 | | 1 (8.3) | 1 (9.1) | 0 | 1 (7.7) | 0 | 1 (7.7) | | 2 (18.2) | 0 | 0 | 0 | 0 | | 1 (7.1) | 1 (11.1) | 2 (25.0) |
| **Chills** |  |  |  |  | |  |  |  |  |  |  | |  |  |  |  |  | |  |  |  |
| Grade 1 | 1 (7.1) | 0 | 0 | 1 (5.9) | | 1 (8.3) | 1 (9.1) | 1 (6.7) | 2 (15.4) | 2 (20.0) | 2 (12.5) | | 1 (9.1) | 2 (33.3) | 0 | 0 | 0 | | 5 (35.7) | 4 (44.4) | 2 (25.0) |
| Grade 2 | 0 | 0 | 0 | 0 | | 4 (33.3) | 1 (9.1) | 0 | 5 (38.5) | 4 (40.0) | 2 (12.5) | | 5 (45.5) | 1 (16.7) | 0 | 0 | 0 | | 3 (21.4) | 1 (11.1) | 1 (12.5) |
| Grade 3 | 0 | 0 | 0 | 0 | | 1 (8.3) | 1 (9.1) | 0 | 1 (7.7) | 0 | 0 | | 3 (27.3) | 1 (16.7) | 0 | 0 | 0 | | 2 (14.3) | 2 (22.2) | 3 (37.5) |

Data are from the dose 1, dose 2, and dose 3 solicited safety sets, which consisted of all participants in the solicited safety set who received dose 1, 2, or 3 and contributed any solicited local or systemic AR data from the time of injection through the following 6 days (dose 1: days 1 through 7; dose 2: month 2 through day 63; and dose 3: month 6 through day 175).

^a^Number of participants in the dose 1, dose 2, or dose 3 solicited safety set.

^b^Number of participants reporting the event.

### Supplementary Table 8. GMRs at Each Timepoint in CMV-Seropositive Participants^a^

|  |  | **mRNA-1647** | | | |
| --- | --- | --- | --- | --- | --- |
| **Timepoint** | **Placebo**  **(n=17)^b^** | **30 µg**  **(n=13)^b^** | **90 µg**  **(n=12)^b^** | **180 µg**  **(n=13)^b^** | **300 µg**  **(n=13)^b^** |
| **nAbs against epithelial cell infection, GMR (95% CI)^c^** | | | | | |
| Month 1 (day 29) | 0.97  (0.83-1.12) | 6.85  (3.68-12.75) | 6.93  (4.25-11.28) | 9.26  (5.49-15.61) | 11.79  (8.14-17.07) |
| Month 3 (day 85) | 0.93  (0.76-1.13) | 14.44  (7.27-28.69) | 9.91  (4.93-19.94) | 19.36  (10.53-35.58) | 17.33  (8.05-37.29) |
| Month 7 (day 197) | 0.96  (0.75-1.24) | 26.23  (12.27-56.08) | 22.40  (9.15-54.84) | 40.83  (16.09-103.61) | 13.38  (6.41-27.92) |
| Month 12 (day 337) | 0.95  (0.75-1.19) | 14.64  (7.61-28.16) | 13.98  (6.19-31.56 | 31.41  (18.09-54.54) | 9.90  (5.07-19.34) |
| Month 18 (day 505) | 1.16  (0.85-1.59) | 12.21  (4.87-30.59) | 12.84  (3.36-49.13) | 14.22  (7.56-26.75) | 6.95  (4.94-9.77) |
| **nAbs against fibroblast infection, GMR (95% CI)^c^** | | | | | |
| Month 1 (day 29) | 0.98  (0.74-1.29) | 2.43  (1.42-4.16) | 2.66  (1.77-4.00) | 2.83  (1.85-4.33) | 2.95  (1.96-4.44) |
| Month 3 (day 85) | 1.03  (0.81-1.32) | 2.48  (1.35-4.57) | 3.00  (1.99-4.54) | 4.08  (2.41-6.90) | 3.77  (2.41-5.89) |
| Month 7 (day 197) | 2.40  (1.41-4.06) | 4.02  (2.41-6.72) | 6.51  (3.77-11.23) | 4.05  (1.69-9.68) | 7.08  (3.85-13.00) |
| Month 12 (day 337) | 3.75  (2.20-6.38) | 8.08  (5.31-12.30) | 5.90  (3.17-10.96) | 6.32  (3.55-11.24 | 7.33  (2.81-19.12) |
| Month 18 (day 505) | 3.28  (2.17-4.94) | 9.50  (4.91-18.36) | 3.11  (1.51-6.42) | 3.65  (1.63-8.15) | 4.86  (1.89-12.48) |
| **Anti-gB antibodies, GMR (95% CI)^c^** | | | | | |
| Month 1 (day 29) | 0.95  (0.87-1.04) | 1.19  (0.99-1.43) | 1.76  (1.44-2.14) | 1.82  (1.47-2.26) | 2.30  (1.66-3.20) |
| Month 3 (day 85) | 0.88  (0.78-0.99) | 1.65  (1.23-2.22) | 2.03  (1.65-2.51) | 2.36  (1.75-3.19) | 2.5  (1.93-3.46) |
| Month 7 (day 197) | 1.01  (0.89-1.16) | 1.93  (1.54-2.42) | 1.89  (1.48-2.41) | 2.04  (1.29-3.23) | 2.00  (1.36-2.92 |
| Month 12 (day 337) | 0.87  (0.78-0.97) | 1.34  (1.00-1.79) | 1.40  (1.05-1.86) | 1.48  (1.23-1.79) | 1.68  (1.25-2.27) |
| Month 18 (day 505) | 1.18  (1.05-1.33) | 1.64  (1.16-2.32) | 1.30  (0.91-1.84) | 1.48  (1.02-2.16) | 1.70  (1.19-2.45) |
| **Anti-pentamer antibodies, GMR (95% CI)^c^** | | | | | |
| Month 1 (day 29) | 0.93  (0.80-1.07) | 7.00  (4.16-11.78) | 8.88  (5.31-14.84) | 11.96  (7.49-19.13) | 12.45  (7.04-22.05) |
| Month 3 (day 85) | 0.95  (0.80-1.13) | 10.54  (5.74-19.36) | 9.86  (5.33-18.23) | 16.12  (12.03-21.61) | 15.11  (6.21-36.81) |
| Month 7 (day 197) | 0.94  (0.78-1.13) | 10.96  (5.32-22.56) | 9.79  (4.94-19.40) | 12.51  (6.88-22.75) | 8.53  (3.59-20.28) |
| Month 12 (day 337) | 0.75  (0.64-0.88) | 4.44  (2.62-7.50) | 4.04  (2.10-7.80) | 5.70  (4.23-7.68) | 4.76  (2.25-10.06) |
| Month 18 (day 505) | 0.90  (0.53-1.54) | 4.14  (1.52-11.28) | 2.28  (0.97-5.38) | 4.95  (2.71-9.03) | 3.74  (2.06-6.79) |

Abbreviations: CMV, cytomegalovirus; gB, glycoprotein B; GMR, geometric mean ratio; nAb, neutralizing antibody.

^a^Data are from the per-protocol set, which was defined by timepoint and consisted of all evaluable participants in the full analysis set who met all eligibility criteria with no major protocol deviations, were administered trial dose according to the protocol, did not receive a concomitant medication or vaccine leading to exclusion from a per-protocol analysis, did not present with a medical condition that might have had an impact on the immunogenicity assessment, complied with the injection schedule, complied with the timing of blood sampling at the corresponding timepoint, had immunogenicity results available for ≥1 assay component at the corresponding timepoint, and were appropriately chracterized by CMV serostatus at screening and baseline.

^b^Number of participants in any per-protocol set.

^c^GMR calculated as postbaseline titer at the respective timepoint/baseline titer.

### Supplementary Figure 1. Overview of the dose escalation, dose selection, and sentinel expansion phases of the trial.


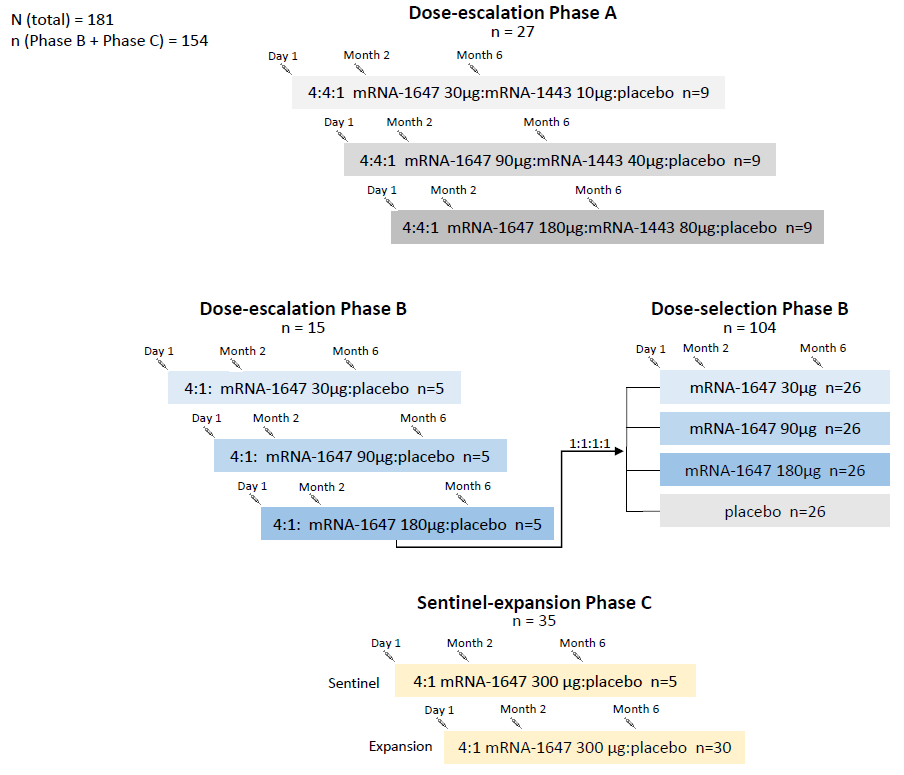


### Supplementary Figure 2. Box plots of antibody-mediated immunogenicity by CMV serostatus.
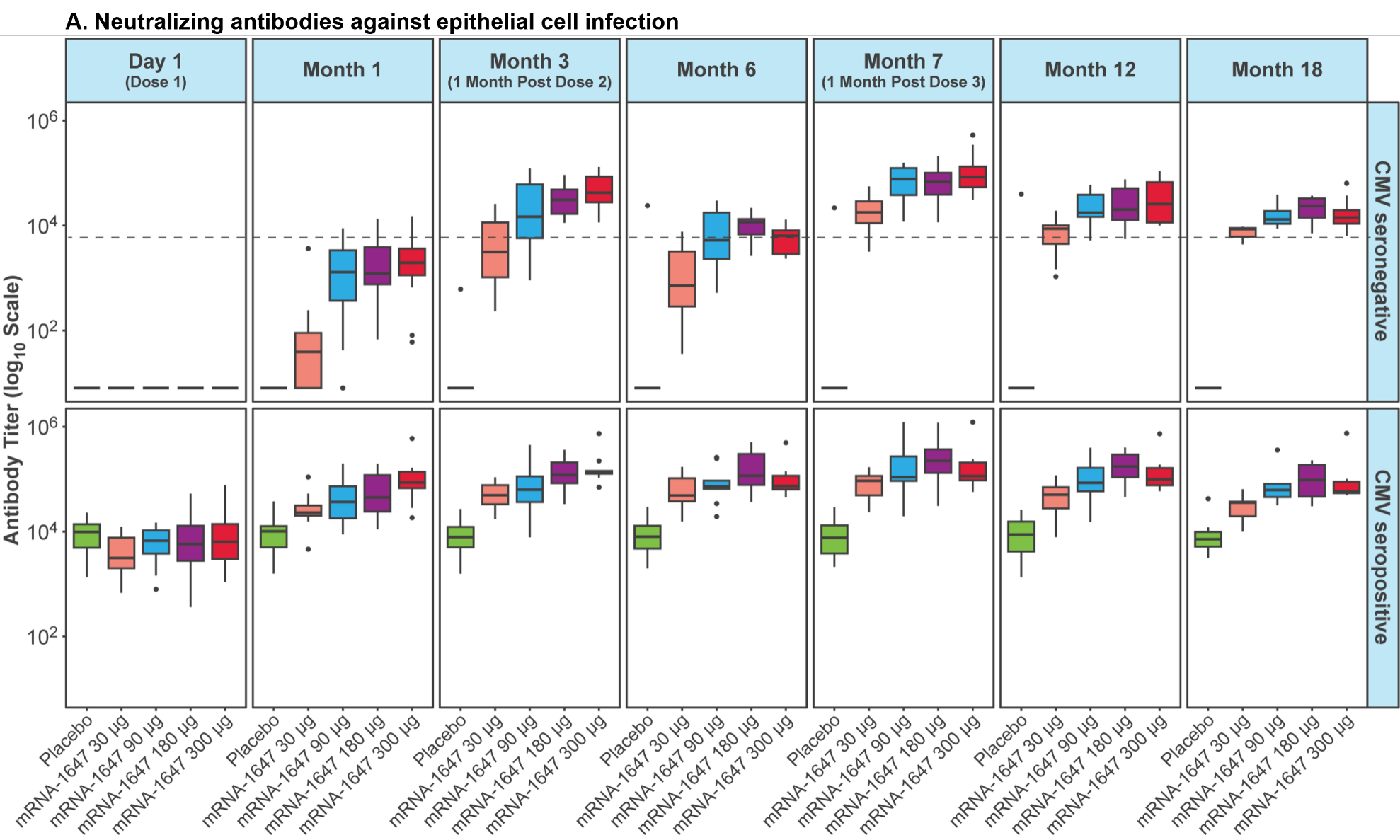


###
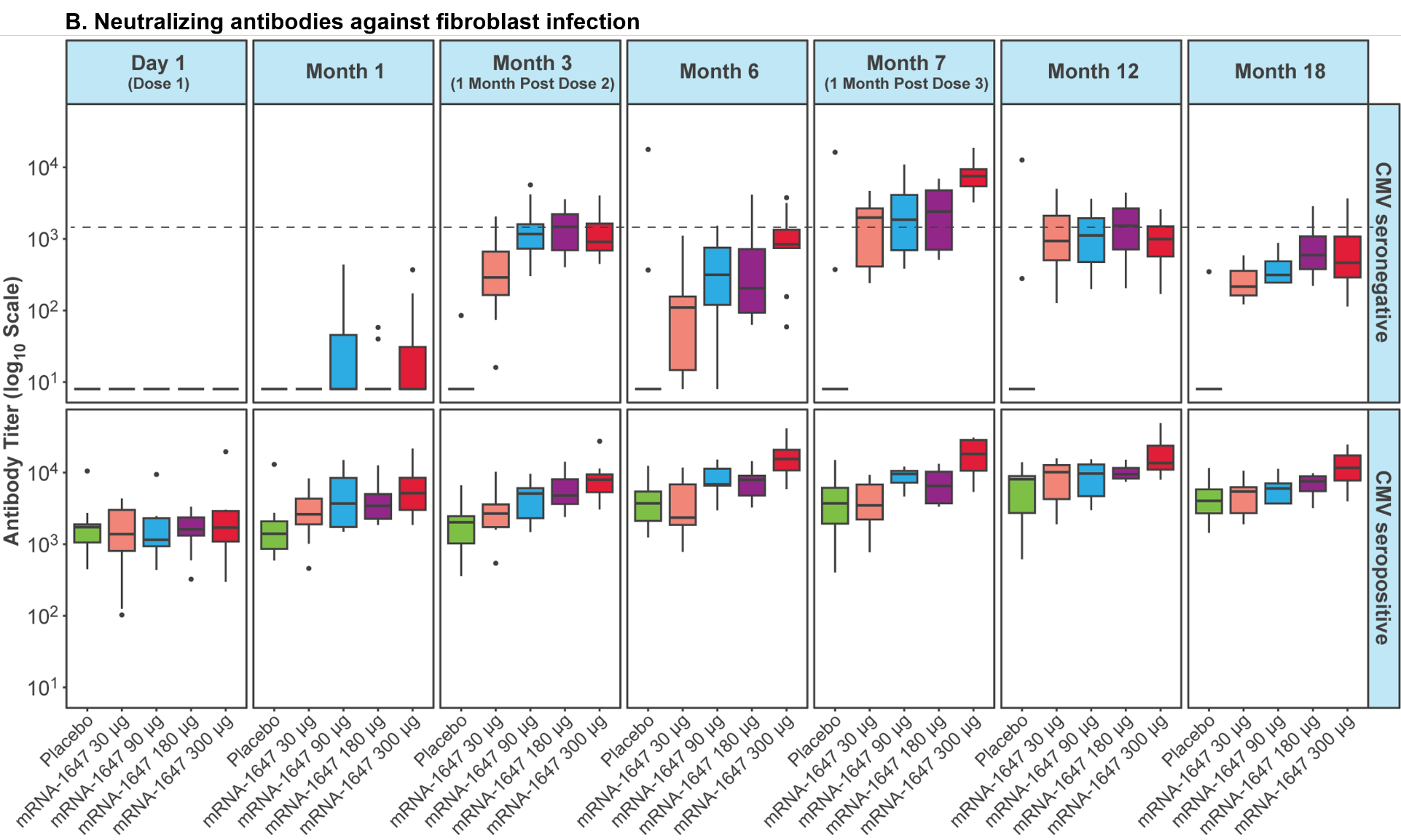


###
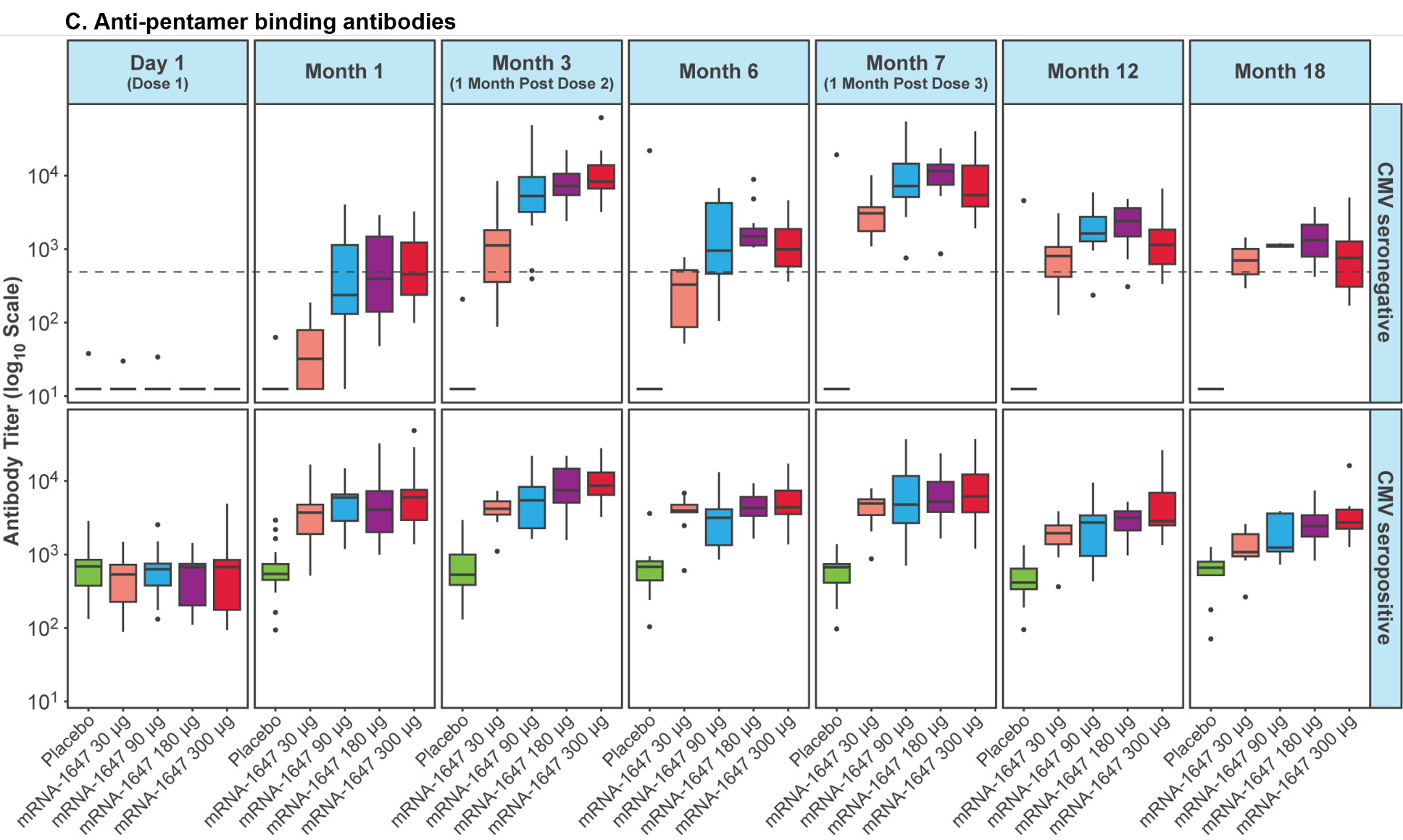


###
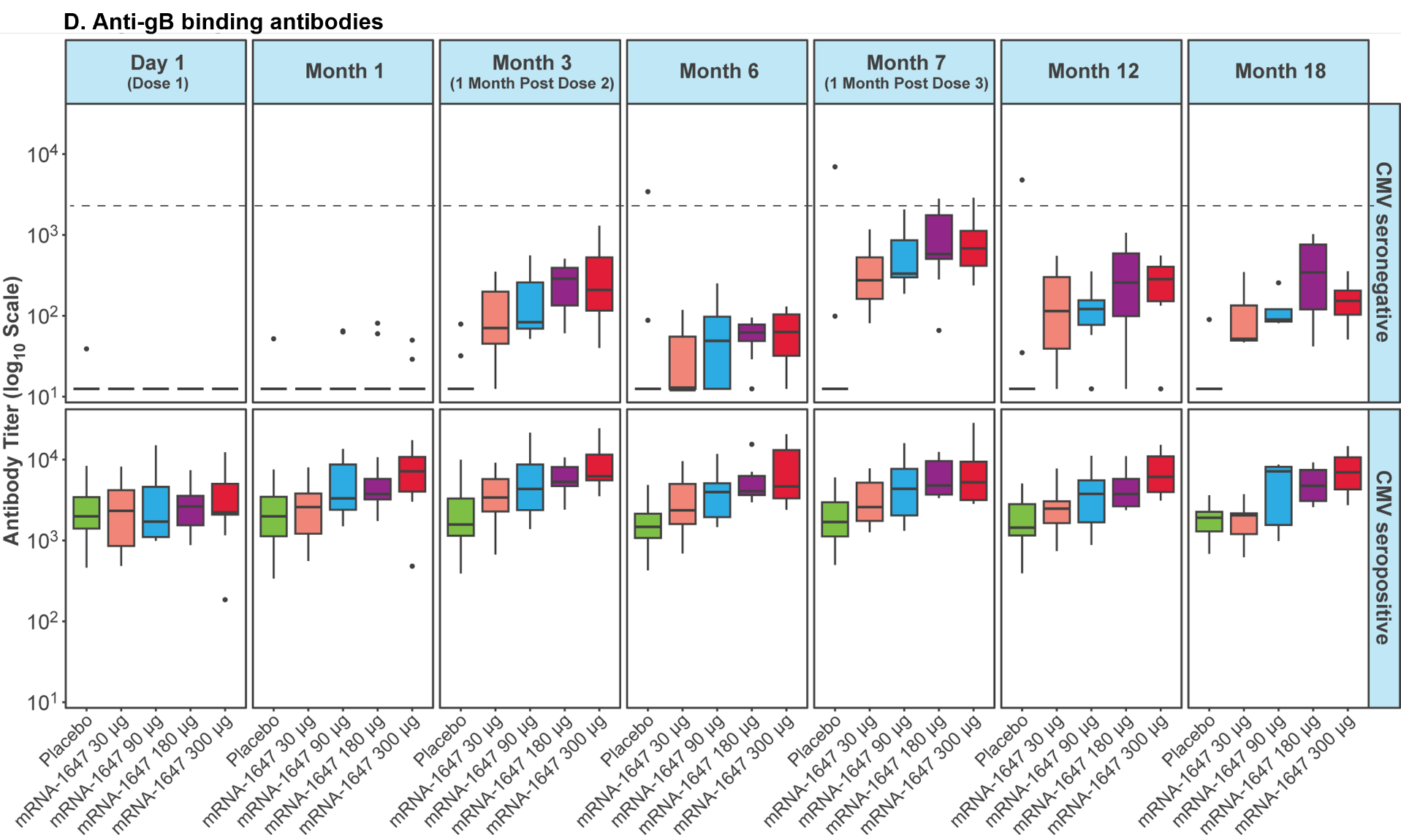


### (A) Neutralizing antibodies against epithelial cell infection, (B) neutralizing antibodies against fibroblast infection, (C) anti-pentamer binding antibodies, and (D) anti-gB binding antibodies are presented using box plots CMV-seronegative and CMV-seropositive participants at Day (Dose1), Month 1, Month 3 (1 Month Post Dose 2), Month 6, Month 7 (1 Month Post Dose 3), Month 12, and Month 18 for placebo (green box) and mRNA-1647 treatment groups (30 µg [peach box], 90 µg [blue box], 180 µg [purple box], and 300 µg [red box]). Dashed line represents the CMV-seropositive baseline GMTs (benchmark). Within each box, horizontal black lines represent median values; boxes extend from the 25^th^ percentile to the 75^th^ percentile of each group’s distribution values; vertical extending lines denote adjacent values; dots denote observations outside the range of adjacent values. Abbreviations: CMV, cytomegalovirus; gB, glycoprotein B; GMT, geometric mean titer.

### Supplementary Figure 3. Antigen-specific cell-mediated immunogenicity by CMV serostatus (change from baseline).


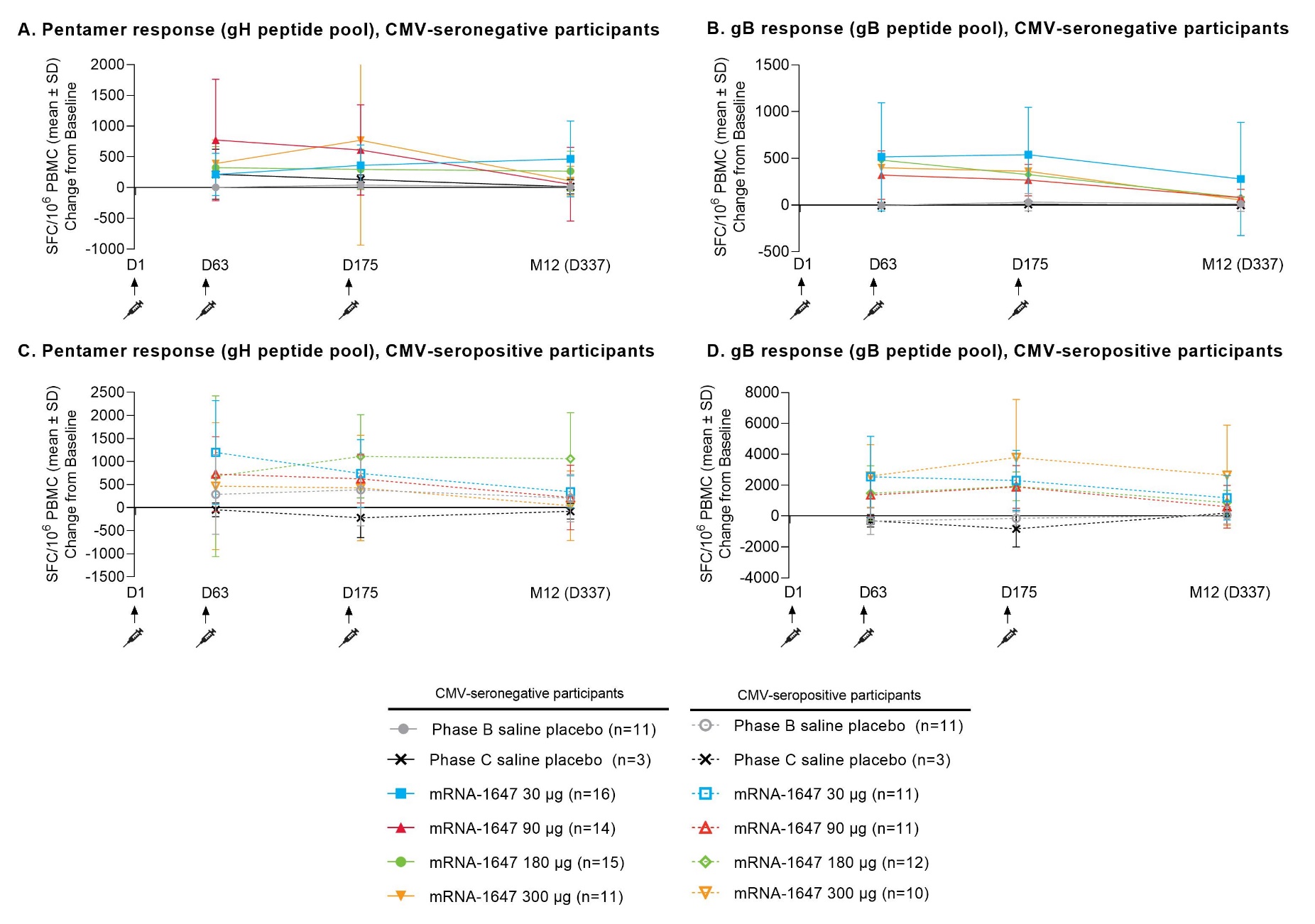


(A) gH peptide pool among CMV-seronegative participants, (B) gB peptide pool among CMV-seronegative participants, (C) gH peptide pool among CMV-seropositive participants, and (D) gB peptide pool among CMV-seropositive participants is presented by treatment group and visit from day 1 through month 12. The mean changes from baseline ± SDs of gH-specific or gB-specific T cells secreting IFN-γ, as determined by IFN-γ ELISpot (reported as SFC/10^6^ PBMCs), for the CMV-seronegative (colored solid lines) and CMV-seropositive (colored dashed lines) participants were plotted over time. Doses 1, 2, and 3 were administered on D1, M2, and M6, respectively, as represented by an arrow and syringe. Placebo results from phases B and C are reported separately by each phase. Data are from the cell-mediated immunogenicity set. n=number of participants in any cell-mediated immunogenicity set. Abbreviations: CMV, cytomegalovirus; D, day; ELISpot, enzyme-linked immunospot; gB, glycoprotein B; gH, glycoprotein H; IFN-γ, interferon-gamma; M, month; PBMC, peripheral blood mononuclear cell; SFC, spot-forming cell.
